# Single-cell multiomics reveals the complexity of TGFβ signalling to chromatin in iPSC-derived kidney organoids

**DOI:** 10.1038/s42003-022-04264-1

**Published:** 2022-11-27

**Authors:** Jessica L. Davis, Ciaran Kennedy, Shane Clerkin, Niall J. Treacy, Thomas Dodd, Catherine Moss, Alison Murphy, Derek P. Brazil, Gerard Cagney, Dermot F. Brougham, Rabi Murad, Darren Finlay, Kristiina Vuori, John Crean

**Affiliations:** 1 UCD School of Biomolecular and Biomedical Science, UCD Conway Institute of Biomolecular and Biomedical Research, University College Dublin, Belfield, Dublin, 4 Ireland; 2 UCD Genomics Core Facility, UCD Conway Institute of Biomolecular and Biomedical Research, University College Dublin, Belfield, Dublin, 4 Ireland; 3 Wellcome-Wolfson Institute for Experimental Medicine, Queen’s University Belfast, BT9 7BL Northern Ireland, UK; 4 UCD School of Chemistry, University College Dublin, Belfield, Dublin, 4 Ireland; 5 Sanford Burnham Prebys Institute for Medical Discovery, La Jolla, CA 92037 USA

**Keywords:** Molecular biology, Stem cells

## Abstract

TGFβ1 plays a regulatory role in the determination of renal cell fate and the progression of renal fibrosis. Here we show an association between SMAD3 and the histone methyltransferase, EZH2, during cell differentiation; ChIP-seq revealed that SMAD3 and EZH2 co-occupy the genome in iPSCs and in iPSC-derived nephron progenitors. Through integration of single cell gene expression and epigenome profiling, we identified de novo ACTA2^+ve^/POSTN^+ve^ myofibroblasts in kidney organoids treated with TGFβ1, characterised by increased SMAD3-dependent *cis* chromatin accessibility and gene expression associated with fibroblast activation. We have identified fibrosis-associated regulons characterised by enrichment of SMAD3, AP1, the ETS family of transcription factors, and NUAK1, CREB3L1, and RARG, corresponding to enriched motifs at accessible loci identified by scATACseq. Treatment with the EZH2 specific inhibitor GSK343, blocked SMAD3-dependent *cis* co-accessibility and inhibited myofibroblast activation. This mechanism, through which TGFβ signals directly to chromatin, represents a critical determinant of fibrotic, differentiated states.

## Abstract (web summary)

Multimodal single-cell analysis on iPSC-derived kidney organoids shows that inhibiting EZH2 attenuates TGFβ1-induced fibrotic gene expression and changes in chromatin accessibility, highlighting a potential therapeutic strategy for renal fibrosis.

## Introduction

Transforming growth factor beta (TGFβ) is a multifunctional regulator, centrally involved in normal homeostasis as well as stemness and regeneration. Dysregulation of TGFβ signalling is implicated in several diseases and inflammatory pathologies; notably in the context of renal fibrosis, TGFβ1 plays a central role as a pro-fibrotic factor and is pivotal for driving the development and progression of end-stage renal disease. TGFβ1 and SMAD2/3 signalling is increased in several experimental animal models^1^ and in patients with kidney disease^2^ and its therapeutic potential confirmed as neutralising antibodies and antisense oligodeoxynucleotides against TGFβ and its receptors attenuate fibrotic responses in multiple models^3–6^.

Transcriptional activation is central to the processes regulating renal cell fate and is modulated by chromatin accessibility at regulatory loci such as promoters and enhancers. Genome-wide approaches, such as DNA-seq and ATAC-seq, mapping dynamic changes in chromatin accessibility during reprogramming to induced pluripotent stem cells (iPSCs), identified that nucleosome occupancy and open chromatin regions are dynamically altered in regulatory regions, especially at the binding sites for reprogramming transcription factors^7,8^. The epigenetic mechanisms that contribute to cellular differentiation and maturation during organ development and in response to altered metabolic states in disease are under intense investigation, as this is widely recognised as a crucial step toward advancing regenerative therapeutics. Central to this process is the polycomb repressive complex 2 (PRC2), a chromatin remodelling complex that mediates silencing of gene expression, yet the identification of polycomb response elements remains elusive^9^. We recently identified an interaction between SMAD3 and EZH2 during stem cell differentiation^10^ that we hypothesise plays a key role in regulating chromatin access in the compromised microenvironment of the fibrotic kidney.

The prevalence of SMAD3 and EZH2 at enhancers and superenhancers strongly suggests a role in the modulation of chromatin access. Superenhancers underlie the identity, lineage commitment and plasticity of stem cells in vivo and are likely to be centrally involved in the determination of cell fate where it has been suggested that they are subject to “super-silencing”, marked by loss of H3K27ac and gain of H3K27me3^11^. Importantly, the dynamics of enhancers and superenhancers during fate determination, for example during wound repair or the acquisition of plasticity, are particularly sensitive to their microenvironment and thus reflect metabolic memory. The coupling of lineage determining factors with SMAD3 and EZH2 to these regulatory regions, is therefore likely to influence the chromatin dynamics required for phenotypic transitions in multiple contexts. Recent breakthroughs highlight the remarkable self-organising ability of pluripotent stem cells to form kidney organoids as a platform for functional, interrogative studies of gene function in development and disease.

Here, we use iPSC-derived kidney organoids to establish a model of cellular differentiation in renal fibrosis. Using multimodal single cell analysis, we show that treatment of organoids with TGFβ1 induced differentiation of resident fibroblasts into myofibroblasts, which was accompanied by the increased expression of fibrosis-associated genes and changes in chromatin accessibility. Inhibition of EZH2 attenuated fibrotic gene expression and TGFβ1-induced changes in chromatin accessibility. The results from this study indicate that the manipulation of the association between SMAD3 and EZH2 may be a useful therapeutic strategy for the resolution of renal fibrosis.

## Results

### Genome-wide localisation of SMAD3 and EZH2 in iPSCs and iPSC-derived nephron progenitor cells

**Fig. 1:**
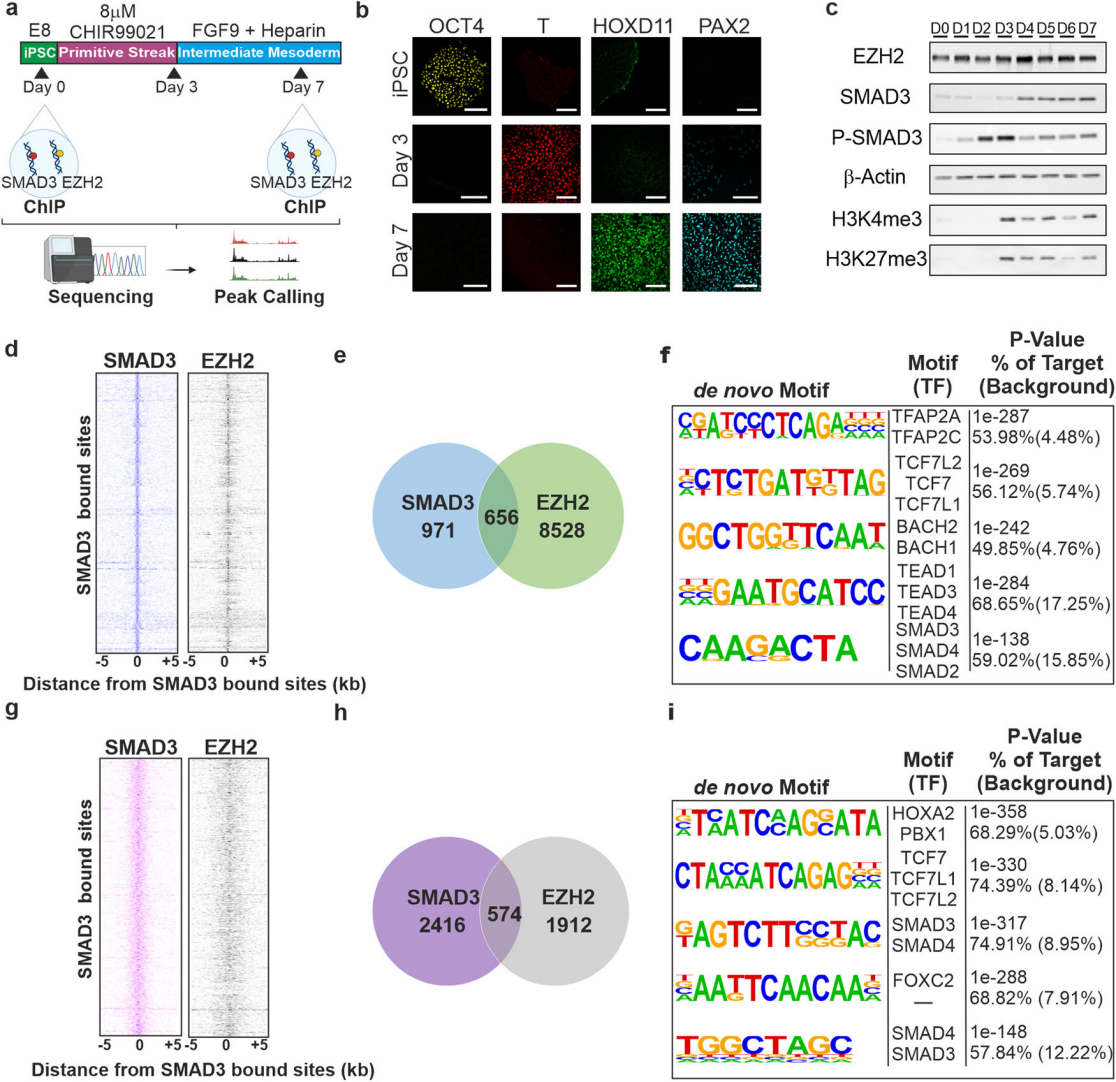
Genome-wide localisation of SMAD3 and EZH2 in induced pluripotent stem cells and iPSC-derived nephron progenitor cells. **a** iPSCs were differentiated towards nephron progenitors using established protocols. Samples for ChIP-seq were taken at day 0 and day 7 of differentiation. **b** Immunostaining of OCT4, T/Brachury, HOXD11, and PAX2 at days 0, 3, and 7. Magnification, ×40. Scale 50 µm. Images are representative of three independent experiments. **c**. Western blots showing expression of EZH2, SMAD3, Phosphorylated SMAD3, H3K4me3, H3K27me3 and β-actin over the course of nephrogenic specification. Blots are representative of 3 independent experiments. **d** SMAD3 and EZH2 co-occupy the genome in iPSCs. Binding plots show the location of SMAD3(left) and EZH2(right) bound sites relative to 971 SMAD3-bound sites. For each SMAD3 bound site (y-axis) the presence of SMAD3 (blue) and EZH2 (grey) sites are displayed within a 10 kb window centred on the SMAD3 bound site. Intensity at position 0 indicates that sites overlap. **e** Venn diagram illustrating the number of SMAD3 and EZH2 bound loci in iPSCs. **f** Motifs enriched and SMAD3 and EZH2 overlapping sites in iPSCs. **g**. SMAD3 and EZH2 co-occupy the genome in NPCs. Binding plots show the location of SMAD3(left) and EZH2(right) bound sites relative to 2416 SMAD3-bound sites. **h** Venn diagram illustrating the number of SMAD3 and EZH2 bound loci in NPCs. **i** Motifs enriched at SMAD3 and EZH2 overlapping sites in iPSCs.

Having previously identified an association between SMAD3 and EZH2 in multiple contexts^10,12^, we first investigated the genome-wide localisation of SMAD3 and the core PRC2 component, EZH2, in human iPSCs and in iPSC-derived nephron progenitor cell (NPC) populations using ChIP-seq. The experiment is summarised as a schematic in Fig. 1a. iPSCs were confirmed as pluripotent, expressing OCT4; cells were differentiated for one week according to^13^ and were positive for the primitive streak marker, T/Brachury, after 3 days of differentiation (Fig. 1b). By day 7, cells were negative for OCT4 and T, while expressing markers of early nephrogenesis such as HOXD11 and PAX2 (Fig. 1b). EZH2 expression was sustained during differentiation whereas SMAD3 was increased during differentiation, coincident with increased H3K4me3 and H3K27me3 (Fig. 1c). ChIP-seq identified 971 SMAD3 peaks and 8228 EZH2 peaks at Day 0, and 2416 SMAD3 peaks and 1912 EZH2 peaks at Day 7. Over 70% of SMAD3 peaks at Day 0 were located at intergenic or distal regulatory regions. Similar data was observed for EZH2. By day 7, 2416 SMAD3 peaks and 1912 EZH2 peaks were apparent with broadly similar genomic distribution (Supplementary Fig. 1). SMAD3 co-occupies the genome with a variety of fate specifying master transcription factors^14^. In stem cells, OCT4 is a SMAD3 target gene and together they form a regulatory circuit to regulate self-renewal; SMAD3 peaks were apparent in iPSCs in the *cis* regulatory enhancer region of the POU5F1 locus, coding for OCT4 (Supplementary Fig. 1).

We further observed that SMAD3 and EZH2 bind similar sites across the genome, confirming their association (Fig. 1d, g). 656 SMAD3 peaks (67.46%) directly overlap with EZH2 peaks in iPSCS (Fig. 1e) while 574 (23.76%) overlap in NPCs (Fig. 1h). Genomic annotation of the overlapping binding sites revealed that most peaks were at intergenic or distal regulatory regions (Supplementary Fig. 1). Using ChromHMM data from H1-ESCs and foetal kidney, we found that overlapping regions in iPSCs and NPCs were mostly enriched for heterochromatin and repressed regions (Supplementary Fig. 1). Motif analysis revealed enrichment of the transcription factors TFAP2A, TCF7, and BACH2/BACH1 in iPSCs (Fig. 1f), and TCF7, PBX1, and FOXC2 in NPCs (Fig. 1i). Regions at both days were expectedly enriched for the SMAD binding element (Fig. 1f, i). By day 7, a similar number of SMAD3 and EZH2 co-occupied sites were apparent (Supplementary Fig. 1b), however we found fewer EZH2 bound sites in the differentiated progenitor population compared to iPSCs, with a concomitant increase in SMAD3 bound sites, likely reflecting SMAD3 binding to open promoters and enhancers in differentiated cells.

### TGFβ1 induces differentiation and activation of fibroblasts in iPSC-derived kidney organoids

The complex heterogeneity of iPSC-derived kidney organoids has led to their proposal as an attractive model for many aspects of renal disease. To generate kidney organoids to model TGFβ responses, we adapted the protocol of^13^ (Supplementary Fig. 2a). Characterisation of kidney organoids by immunocytochemistry and transmission electron microscopy is outlined in Supplementary Fig. 2 and is comparable to other published kidney organoids^13^. We performed single cell RNA-sequencing (scRNAseq) to transcriptionally validate and characterise the heterogenous populations of cells within control organoids and those treated with TGFβ1. To identify the cell types within each of the organoids, clusters from the control organoid were used for annotation (Supplementary Fig. 3a, b) and the top differentially expressed genes from each cluster were compared to known markers of the developing kidney^15–17^ as well as marker genes from published kidney organoid protocols^18–21^ and the Human Nephrogenesis Atlas^22^ (Supplementary Data 1 and 2). We observed some non-kidney cell populations in the organoid, consistent with single cell data from kidney organoids generated using the Takasato protocol^18,19^. RNA velocity and Phate-based trajectory analysis revealed that Cluster 3 (Nephron progenitors) was most likely the parental cluster of cells for Cluster 0 (Fibroblast 1; Fib1), the PDGFRA^+ve^ Cluster 1 (Fibroblast 2; Fib2), and Cluster 2 (Proliferating Fibroblast) (Supplementary Fig. 3c). Additionally, Cluster 5 (Podocyte/SSB/PT) originated partially from these three clusters. The muscle progenitor population (Cluster 10) originated from a portion of Cluster 0 (Stroma 1; S1).

**Fig. 2:**
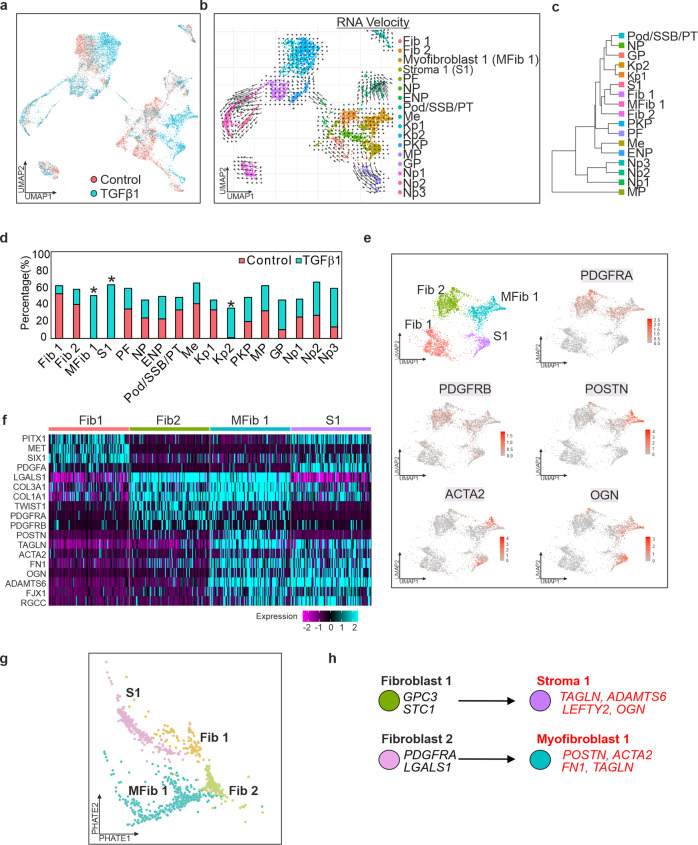
Single-cell RNA-seq characterisation of iPSC-derived kidney organoids after treatment with TGFβ1. **a** UMAP projection of 8176 single cells (4119 Control/4,057 TGFβ1) revealing 17 distinct clusters in iPSC-derived kidney organoids, including new distinct populations of stromal and muscle like cells in response to TGFβ1. Each dot represents a single cell, colour coded for control (red) and TGFβ1 (red). **b** RNA Velocity Map illustrating altered trajectories of cells in organoid in response to TGFβ1. Long arrows correspond to changes in gene expression and are undergoing differentiation while short arrows represent terminally differentiated cells. **c** Cluster tree illustrating relationship between new clusters. **d** Three new populations of cells were apparent in response to TGFβ1 and annotated as MFib1, S1 and Kp2, corresponding to differentiating myofibroblast-like and epithelial populations, respectively. **e** Expression of *PDGFRA, PDGFRB, POSTN, ACTA2*, and *OGN* in Fib1-2 and MFib1, and S1. **f** Heatmap of selected marker genes used to annotate the MFib1 and S1 clusters. **g** Cell Phate Map illustrating fate trajectories of Fib 1, Fib 2, MFib1, and S1 within the organoid. **h** Lineage tree of the fibroblast and stromal clusters in response to TGFβ1.

To identify the cell types within TGFβ1 treated organoids, 4057 cells were integrated and clustered together with the untreated organoid. Two distinct populations were induced by TGFβ1 (Fig. 2d), one of which bears similarity to the single cell data of pericyte and myofibroblast populations suggested by others^23^ as the major sources of matrix production in chronic kidney disease (Supplementary Fig. 4a, Supplementary Data 3). Cluster 2 (POSTN^+ve^, PDGFRA^+ve^, PDGFRB^+ve^, ACTA2^+ve^) readily identify as activated myofibroblasts whereas the second population, Cluster 3 (VIM^+ve^, COL1A1^+ve^, PDGFRA^−ve^, ACTA2^+ve^) were less easily identifiable but the expression of *VIM* and *COL1A1* indicated that these cells were of a stromal lineage (Fig. 2e and Supplementary Fig. 4b). Interestingly, TGFβ1 also induced the expansion of a population of epithelial-like cells (Kidney Progenitor 2) cluster by 34.3% (Fig. 2d); these cells did not appear to be actively proliferating, nor did they exhibit any signs of epithelial to mesenchymal transition.

The PDGFRA^+ve^ Myofibroblast 1 (MFib1) cluster was marked by the increased expression of periostin (*POSTN*), transgelin (*TAGLN*), fibronectin (*FN1*), and α-smooth muscle actin (*ACTA2*) indicating that this cluster was mostly comprised of myofibroblast-like cells^23^. Cluster S1 was marked by increased expression of *FST*, *LEFTY2*, *ADAMTS6*, *OGN*, and *SULF2*. Both clusters expressed several common genes, most notably several extracellular matrix (ECM) proteins associated with fibrosis including *FN1, COL1A1, COL1A2, COL3A1, COL22A1*, and the collagen crosslinking enzyme *LOX* (Supplementary Data 1) as well as fibroblast genes *CALD1, PALLD, NCAM1* (Supplementary Fig. 4b). Cells in both clusters were negative for the smooth muscle genes, *CNN1* and *MYH11* (Supplementary Fig. 4c). Analysis of all marker genes in control and TGFβ1 treated organoids revealed striking similarity with Fib1 and Fib2, suggesting that these cells had differentiated in response to TGFβ1 (Fig. 2c, f); Phate trajectories and RNA velocity analysis similarly suggested that Fib2 was the parent cluster of MFib1 while Fib1 was the parent cluster of S1 (Fig. 2b, c, g, h). TGFβ target gene and core matrisome gene scores were significantly higher in clusters MFib1 and S1 (Supplementary Fig. 5a, b).

**Fig. 3:**
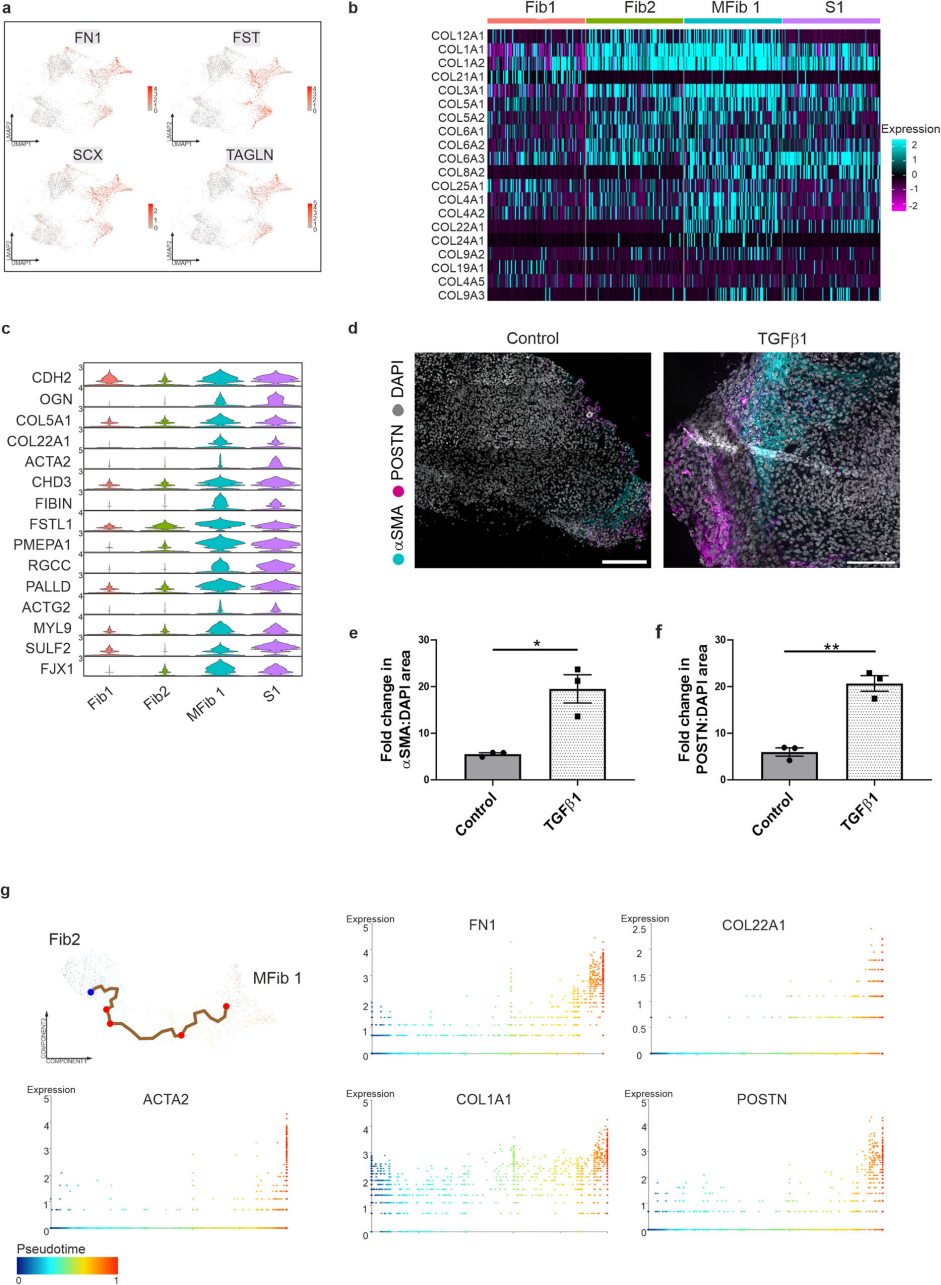
TGFβ1 induces the differentiation of stromal clusters and activation of fibroblasts. **a** UMAPs of differentially upregulated genes in TGFβ1-treated organoids. **b** Scaled expression of collagens in Fib1-2 and MFib1, and S1. **c** Violin plots of differentially expressed genes in MFib1 and S1 compared to parent populations Fib1-2. **d** Organoids were treated with TGFβ1 for 48 h. TGFβ1 induced expression of α-smooth muscle actin (αSMA) and periostin (POSTN) in kidney organoids relative to control Scale bar: Control, 200 µm, TGFβ1, 150 µm. Images are representative of three independent experiments. **e** αSMA/DAPI area and **f** POSTN/DAPI area in untreated or TGFβ1 treated organoids. Each symbol represents the mean of 18 randomly imaged fields, taken from one organoid per condition, from three independent experiments. Data are presented as the mean ± SEM. *P ≤ 0.01, **P ≤ 0.0015. **g** Single-cell trajectory analysis plots of gene expression changes for *ACTA2, FN1, COL22A1, COL1A1*, and *POSTN* in Fib2 and MFib1. Cells are coloured by pseudotime.

Differential gene expression (DEG) analysis was performed to understand the variation between the newly formed stromal clusters and their parent clusters. 491 genes were increased in MFib1 compared to its parent cluster Fib2 (Supplementary Data 4); 349 genes were significantly increased in S1 compared to its parent cluster Fib1. In response to TGFβ1, MFib1 and S1 had increased expression of *ACTA2*, *POSTN*, *FN1*, *FST, TAGLN*, *SCX*, *CDH2, OGN*, and several collagens (Fig. 3a–c and Supplementary Data 4). Expression of *OGN, COL22A1, TAGLN, FIBIN*, *RGCC, ACTG2*, and *ACTA2* were exclusive to the new clusters and are generally accepted marker genes of fibroblast-to-myofibroblast transition^23–25^ (Fig. 3c, g). Of note, immunofluorescence confirmed significantly increased staining for αSMA and periostin in interstitial/stromal cells in response to TGFβ1 (Fig. 3d–f). In addition, elevated picrosirius red staining was observed in response to TGFβ1 (Supplementary Fig. 6a, b). Several genes associated with myofibroblasts were also highly expressed in MFib1 compared to Fib1-2 and S1 such as *POSTN, FGF18, MGP*, and *BGN* (Supplementary Fig. 7a). Protein network maps for differentially upregulated genes in MFib1 revealed a tight protein-protein interaction (PPI) network significantly associated with the biological processes of ECM organisation, organ development, and morphogenesis (Supplementary Fig. 7b). In S1, expression of *LEFTY2, PAX7, DLK1*, and *SYT6* was observed compared to the other stromal clusters (Supplementary Fig. 7c). PPI’s for differentially upregulated genes in S1 were significantly associated with developmental processes and cell differentiation (Supplementary Fig. 7d).

Differential expression analysis also revealed that 112 genes were downregulated in MFib1 compared to Fib2 and these genes included *ID3, ID1, NR2F1*, and *VEGFD* (Supplementary Fig. 8a). In S1, 176 genes were differentially downregulated compared to Fib1 and these genes included *MET, EDN3, IGDCC3*, and *ST1* (Supplementary Fig. 8c). PPIs for genes differentially downregulated in MFib1 and S1 in response to TGFβ1 were enriched for the GO terms associated with DNA replication, organic substance biosynthetic processes, and epithelial development (Supplementary Fig. 8c, d). In summary, TGFβ1 treatment of kidney organoids induces differentiation of stromal clusters, activation of fibroblasts, and stimulates fibroblast-to-myofibroblast transition.

Next, we aimed to assess whether genes upregulated in response to TGFβ1 were also associated with renal fibrosis. Genes upregulated within MFib1 and S1 in response to TGFβ1 were compared with publicly available bulk RNA-seq data from the tubulointerstitium of healthy living donors (*n* = 9) and patients with renal fibrosis (*n* = 10; www.nephroseq.org). A significant decline in glomerular filtration rate (GFR) confirmed that patients had developed kidney disease (Supplementary Fig. 9a). A large proportion of upregulated DEGs were also significantly increased in fibrotic kidney (*n* = 10) compared to healthy living control (*n* = 9; Supplementary Fig. 9a, b). This indicates that TGFβ1 treatment of kidney organoids induces a similar fibrotic response to that observed in vivo.

### GSK343 attenuates αSMA and periostin expression in TGFβ1 treated kidney organoids

**Fig. 4:**
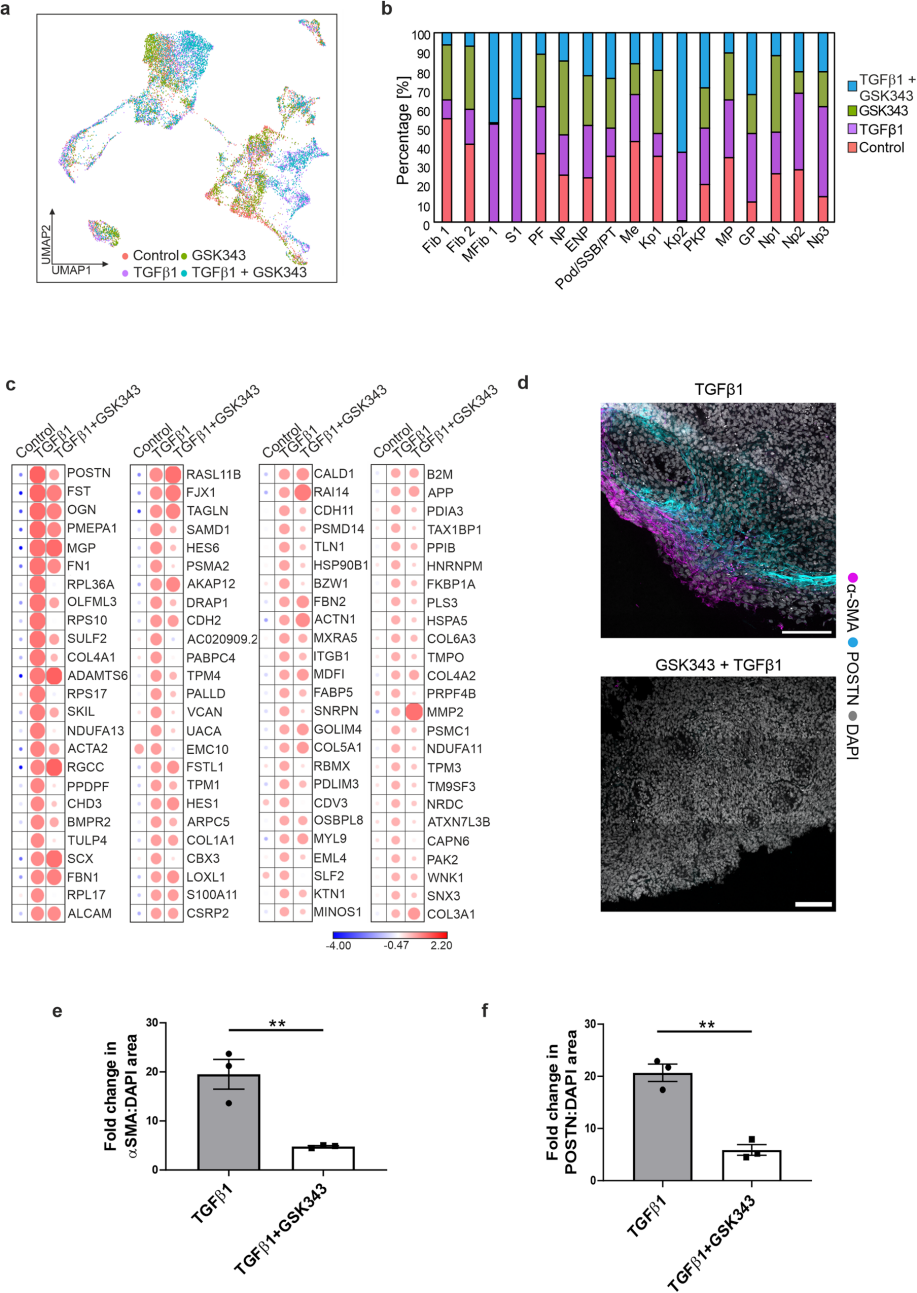
GSK343 attenuates a subset of TGFβ1-induced fibrotic gene expression. **a** Integrated UMAP of scRNAseq data for all conditions. **b** Bar chart depicting the percentage of cells per cluster in each sample. **c** Scaled gene expression for MFib1 in control, TGFβ1, and TGFβ1 + GSK343 treated organoids. **d** Organoids were pre-treated with the EZH2 inhibitor GSK343 for 1 h prior to treatment with TGFβ1 for 48 h. Immunostaining of αSMA and periostin (POSTN) in TGFβ1−, and TGFβ1 + GSK343-treated organoids. Scale bar 150 µm. Images are representative of three independent experiments. **e** αSMA/DAPI area and **f** POSTN/DAPI area in TGFβ1 and TGFβ1 + GSK343 treated organoids. Each symbol represents the mean of 18 randomly imaged fields, taken from one organoid per condition, from three independent experiments. Data are presented as the mean ± SEM. **P ≤ 0.01 (αSMA/DAPI area = *P* ≤ 0.0083; POSTN/DAPI area = *P* ≤ 0.0017).

Current evidence suggests that EZH2 likely regulates activation of fibrogenic gene transcription by interacting with the TGFβ1 signalling pathway^26–29^. Given our previous observations on SMAD3 and EZH2, we hypothesised that targeting this interaction might change the response of the stromal populations to TGFβ1. Kidney organoids were treated with the selective and highly potent S-adenosyl-L-methionine competitive EZH2 inhibitor, GSK343^30^. We mapped the single cell transcriptome of kidney organoids treated with GSK343 and TGFβ1. During processing, low-quality cells were removed and 3584 cells for GSK343 and 3,823 cells for TGFβ1 + GSK343 were integrated and clustered together with the control and TGFβ1 organoids as previously described. Kidney organoids treated with GSK343 alone served as a control and single cell analysis revealed that these organoids were very similar to the control organoids with no apparent cell differentiation or significant changes in cell number per cluster (Fig. 4a, b). Clusters in TGFβ1 + GSK343 organoids mapped very well to TGFβ1 and the number and proportion of cells within each cluster was extremely similar (Fig. 4a, b). In addition, the proliferation of the Kidney Progenitor 2 cluster was also evident in TGFβ1 + GSK343 organoids. At the gene expression level, pre-treatment of organoids with GSK343 before TGFβ1 treatment did not change the identity of the MFib1 and S1 clusters (Fig. 4a, c); however, downregulation of several myofibroblast associated genes (*ACTA2, POSTN, COL4A1*, and *SULF2*) in the PDGFRA^+ve^ MFib1 cluster was apparent (Fig. 4c). In addition, immunofluorescence analysis revealed that pre-treatment with GSK343 ablated the increased expression of αSMA and periostin within the interstitial/stromal cells of the kidney organoids in response to TGFβ1 (Fig. 4d–f). Overall, we show that inhibition of EZH2 in kidney organoids attenuates the expression of genes associated with kidney fibrosis.

### Chromatin accessibility is dynamically regulated by TGFβ1 and EZH2 in iPSC-derived kidney organoids

Transcriptional responses to TGFβ1 are to a large degree shaped by the interaction of activated SMAD proteins with chromatin. TGFβ superfamily members exert their transcriptional control in part through the regulation of enhancer activity and through the direct interaction of SMAD complexes with promoters. To further probe the relationship between TGFβ1 and EZH2 at the chromatin level, we employed single cell ATAC-seq (scATACseq) to map genome accessibility within stromal cells during fibroblast-to-myofibroblast transition in iPSC-derived organoids exposed to TGFβ1 in the presence or absence of GSK343.

**Fig. 5:**
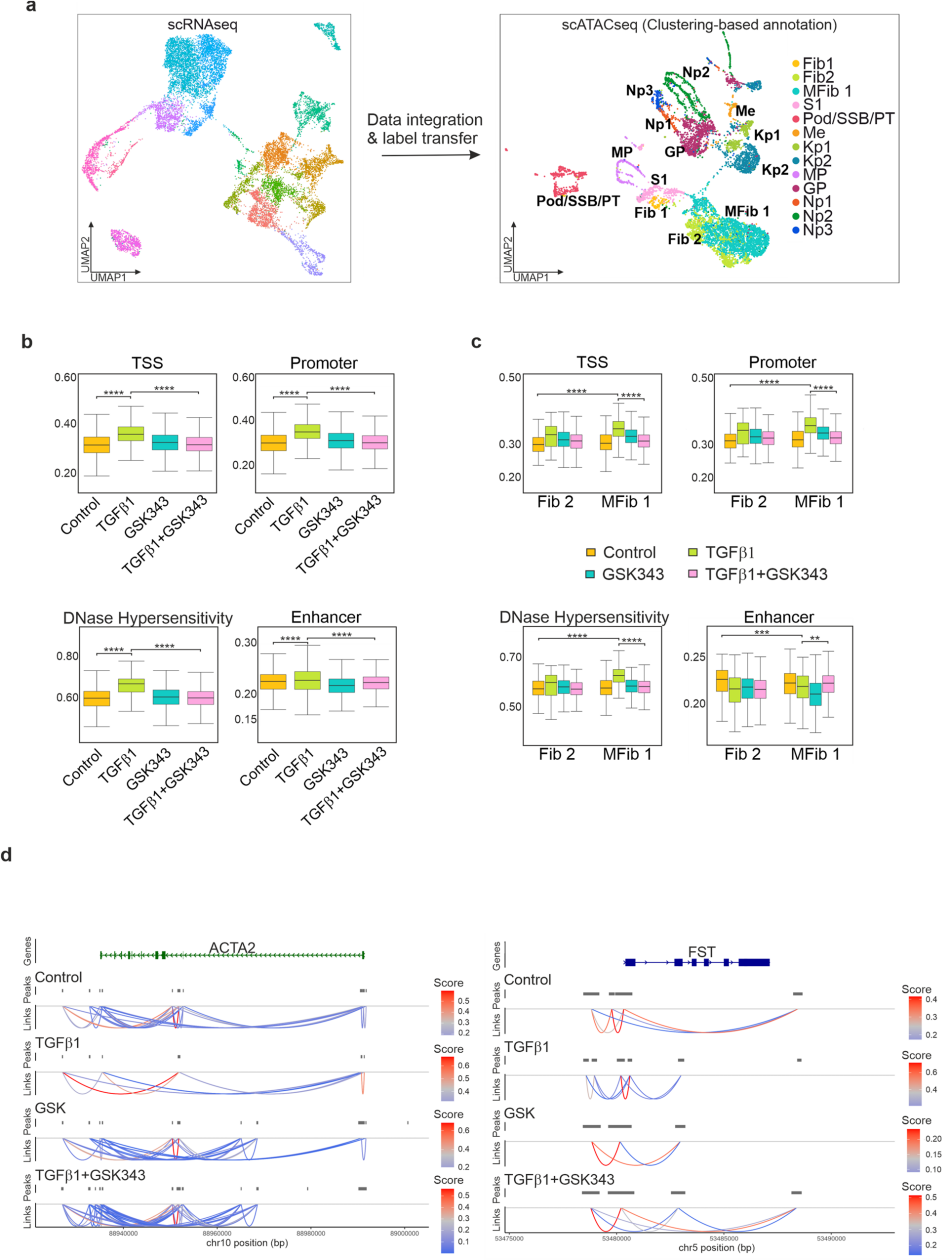
Integration of single cell RNA-seq and ATAC-seq identifies open chromatin regions and increased accessibility in response to TGFβ1. **a** Multi-omics integration strategy for processing the scATACseq dataset. Annotated clusters in the scRNAseq dataset were used to predict cell types in the scATACseq dataset. UMAP plot of scATACseq dataset with gene activity-based cell type assignments. **b** Global changes in chromatin accessibility at Transcription Start Site (TSS), promoters, enhancers, and DNase I hypersensitivity sites. ****P ≤ 0.0001, pairwise *t*-test with Bonferroni correction. **c** Changes in chromatin accessibility at Transcription Start Site (TSS), promoters, enhancers, and DNase I hypersensitivity sites in response to TGFβ1 in Fib2 and MFib1 is inhibited by GSK343. **P ≤ 0.01; ***P ≤ 0.001; ****P ≤ 0.0001; pairwise *t*-test with Bonferroni correction. **d** Prediction of *cis* co-accessibility networks (CCAN) at sample loci in response to TGFβ1 and GSK343. Higher co-accessibility score (red) indicates higher co-accessibility between promoter and enhancer elements.

Using multimodal integration, we characterised the cell types present in our scATACseq dataset by comparing chromatin accessibility profiles to gene expression. Briefly, to interpret the scATACseq clusters, we used the annotated scRNAseq dataset to predict the cell types present within the scATACseq dataset. Annotation of the scATACseq clusters was performed by creating a gene-activity matrix using a measure of chromatin accessibility within the gene body and promoter of protein-coding genes. A set of integration anchors were identified between the scRNAseq dataset and the gene-activity matrix which allowed for the prediction and assignment of cell types within the scATACseq dataset. Following integration, labels were transferred from the annotated clusters in the scRNAseq dataset to the predicted clusters in the scATACseq dataset (Fig. 5a); Gene activity at promoters was generally a reliable predictor of gene expression (Supplementary Fig. 10a, b) and the key marker genes chosen for cluster annotation shared correlation patterns across the datasets.

To assess global changes in chromatin accessibility, fragments were counted and scored based on their proximity to genomic locations such as the Transcription Start Site (TSS), promoters, enhancers, and DNase I hypersensitivity sites. TGFβ1 treatment increased global accessibility at the TSS, promoters, and DNase I hypersensitivity sites (Fig. 5b and Supplementary Data 5). Significant changes were observed at enhancers in response to TGFβ1 and pre-treatment with GSK343 prior to TGFβ1 generally prevented these accessibility changes (Fig. 5b). To assess changes in chromatin accessibility between the myofibroblast cluster, MFib1, and its parent cluster, Fib2, we scored the number of fragments present at the TSS, promoters, enhancers, and DNase I hypersensitivity sites (Fig. 5c, Supplementary Data 6). In response to TGFβ1, accessibility was significantly increased in both cell clusters at promoters, TSS and DNase I hypersensitivity regions. Conversely, a decrease in accessibility was noted at enhancers within MFib1. Again, pre-treatment with GSK343 prior to TGFβ1 significantly prevented these accessibility changes (Fig. 5c).

We next investigated differences in chromatin accessibility between MFib1 and Fib2 using Signac. 10,678 Differentially Accessible (DA) regions were identified in MFib1 while 10,222 regions were identified in Fib2 (Supplementary Data 7). Chromatin accessibility at *cis* regulatory elements such as enhancers and promoters signify reduced nucleosome density indicating the binding of sequence specific transcription factors. Regulatory elements tend to cluster to form co-accessibility networks that regulate gene expression^31^. To better understand the mechanism by which TGFβ1 controls gene expression at distal elements, we used Cicero to predict *cis* regulatory DNA interactions in control, TGFβ1, and TGFβ1 + GSK343 treated organoids for differentially expressed genes identified in MFib1. Differentially upregulated genes in MFib1 compared to Fib2 included αSMA (*ACTA2*) and follistatin (*FST*). For both these genes, TGFβ1 treatment changed co-accessibility between enhancer and promoter sites, whereas pre-treatment with GSK343 reversed this increase in a similar manner to that observed in control (Fig. 5d). These results indicate that EZH2 may be required for TGFβ1 mediated changes in chromatin co-accessibility and enhancer-promoter interactions.

*Cis* co-accessibility networks are families of chromatin regions that can be used to predict looping interactions between regulatory elements that are likely to be located in close proximity to one another^31^. We next investigated whether TGFβ1 increased co-accessibility links between regulatory elements at putative enhancer locations identified to be occupied by both SMAD3 and EZH2 in our iPSC and NPC ChIP-seq experiments. In both IPSCs and NPCs, TGFβ1 increased regulatory interactions between distal elements up- and down-stream of the putative enhancer regions. Connections between these presumed co-accessible regions were not observed in control or in organoids pre-treated with GSK343 (Supplementary Fig. 11).

**Fig. 6:**
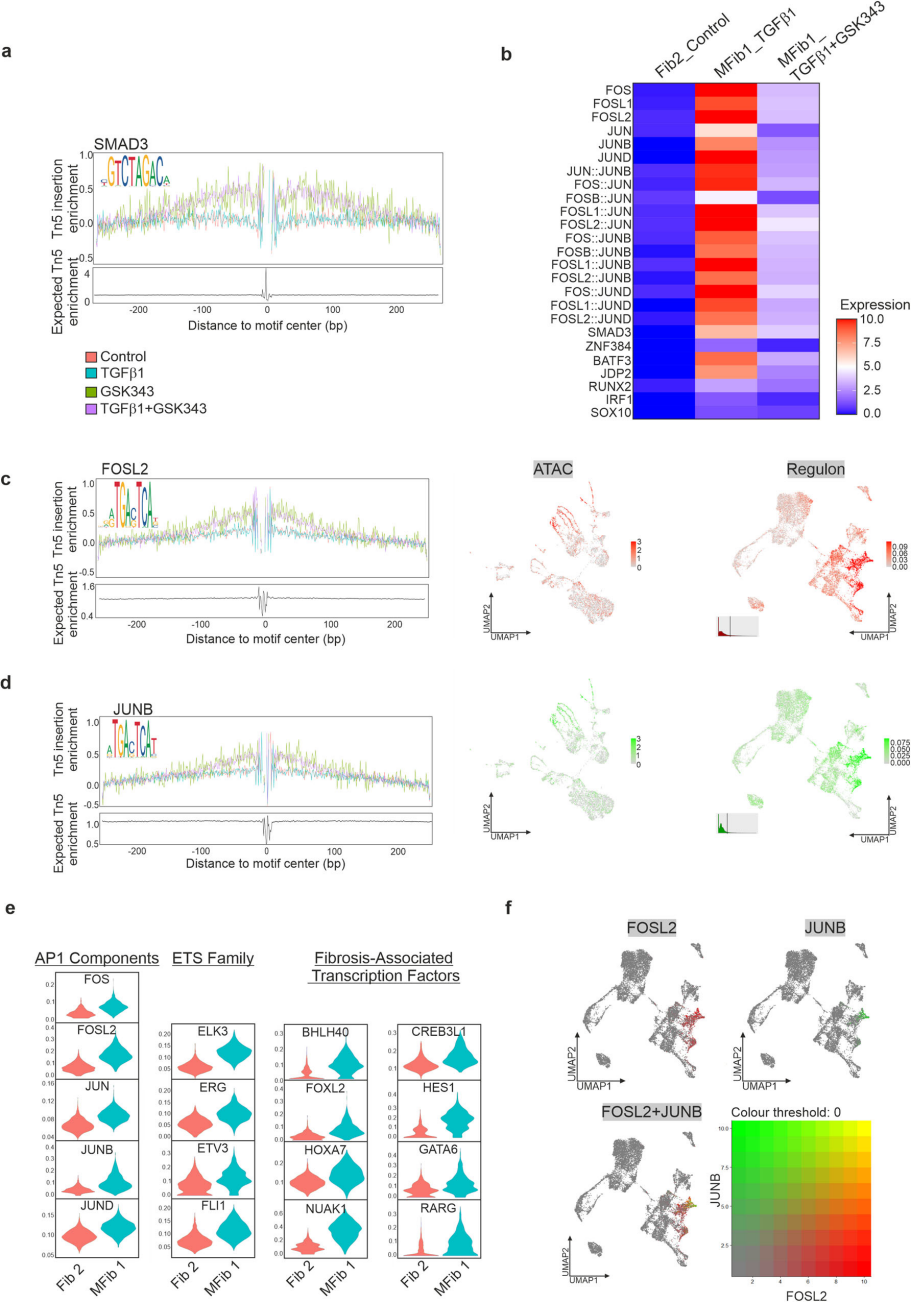
De novo clusters in response to TGFβ1 represent a “fibrotic” regulon, enriched with motifs for SMAD3 and Fos/Jun. **a** Motif-centric footprinting showing enrichment for SMAD3. **b** Motif activity at accessible regions in MFib1 treated with TGFβ1 or TGFβ1 + GSK343, compared to Fib2 (Control). AP1 motif enrichment in myofibroblasts is decreased by inhibition of EZH2. **c**, **d** Motif-centric footprinting showing enrichment for factors associated with a “fibrotic” regulon; Shown are representative SCENIC-UMAPs of the regulons from the top enriched motifs (right hand panels), and the correspondence with scATACseq enriched motifs (centre panels). **e** Differentially regulated transcription factor networks (“regulons”) associated with AP1 complex, ETS family, and other fibrosis-associated transcription factors in MFib1 compared to its parent cluster Fib 2. **f** Blended UMAPs of transcription factor regulon for FOSL2 and JUNB identified by SCENIC in MFib1 and S1.

Enhancers and promoters can associate via long-range interactions and this is partially regulated by transcription factors^32^. We used chromVAR to predict transcription factor ‘activity’ based on the presence of binding motifs for differentially accessible regions identified within Fib2 and MFib1. We observed increased motif activity for SMAD3, and members of the AP1 family such as JUNB and FOSL2 (Fig. 6a, b), that were exclusive to differentially accessible regions identified in MFib1 in response to TGFβ1. Treatment with GSK343 attenuated this activity (Fig. 6b). Increased footprint depth was also observed for SMAD3, FOSL2, and JUNB (Fig. 6a, c, d). We next employed SCENIC (Single-Cell rEgulatory Network Inference and Clustering), which defines core transcription factors with their positively regulated target genes in single cells, to investigate this regulatory network in the scRNAseq data. Previous studies have identified AP1 as a core modulator of TGFβ activity^33–35^. We similarly identified high AP1 regulon activity in MFib1 in response to TGFβ1 (Fig. 6e, f and Supplementary Data 8), among several novel regulons including ETS family members and other fibrosis-associated transcription factors (Fig. 6e and Supplementary Data 8**)**. This establishes that the TGFβ1 response requires the AP1 regulon for fibroblast-to-myofibroblast transition in kidney organoids and identifies a transcriptional regulatory mechanism centred on the ETS family and several fibrosis-associated transcription factors including NUAK1, CREB3L1 and RARG constituting a new regulatory hierarchy.

## Discussion

The biochemical processes that contribute to the initiation and progression of renal fibrosis are multi-factorial in nature and consist of the complex interplay between numerous metabolic and growth factor signalling pathways. At present, intense efforts have been dedicated to target pathogenic mediators of renal fibrosis such as oxidative stress, inflammation, AGEs, and growth factors such as TGFβ1, PDGF, and CTGF (reviewed in ref. ^36^). Some of these strategies have been successful in delaying progression in human clinical trials but most have not restored kidney function.

This study aimed to model TGFβ1-induced renal injury using iPSC-derived kidney organoids. A major limitation of this model is organoid immaturity, for example, the population of podocyte-like cells within the organoid are at best immature precursors; although they stained positive for WT1, positive staining for slit diaphragm markers like nephrin and podocin was somewhat equivocal. While acknowledging that the organoids represent immature embryonic-like tissues nevertheless, the major findings from this study were subsequently validated by multiple modalities. Within the organoid, three novel clusters were identified in response to TGFβ1 corresponding to two differentiating stromal populations and a differentiating epithelial progenitor population. In fibrosis, TGFβ1-induced epigenetic modifications such as DNA methylation and post-translational histone modifications are required to establish and maintain the persistent activation of fibroblasts^37,38^. In addition, increasing evidence suggests that abnormal expression of EZH2 is associated with enhanced fibrosis and contributes to the pathogenesis of renal disease^27,39^. SMAD3 is key to regulating cell fate during the maintenance of pluripotency as well as during differentiation in development and disease^14,40^. It is now well established that SMAD3 cooperates with EZH2 to mediate cell fate decisions in retinal epithelial cells, neuron progenitors, embryonic stem cells, and in activated fibroblasts^10,12,39,41^. We demonstrated that SMAD3 and EZH2 co-localise at putative enhancers and heterochromatin regions in iPSCs and in iPSC-derived nephron progenitor cells. SMAD3 is important for the maintenance of self-renewal through cooperation with core pluripotency factors and co-repressors^42,43^. In mouse ESCs, Smad3 and Oct4 interact with PRC2 to maintain the repression of Rif1 to promote genomic stability^44^. In our ChIP-seq data, regions co-occupied by SMAD3 and EZH2 in iPSCs were largely enriched for co-repressors such as TEAD and BACH2. We propose that SMAD3 is a master regulator of cell fate and cooperates with EZH2 to facilitate and dynamically maintain chromatin states to preserve pluripotency in human iPSCs. SMAD3 is also thought to participate in maintaining the self- renewal of iPSC-derived NPCs^45^ and in our ChIP-seq data, SMAD3 and EZH2 co-occupied sites originally presumed to be repressive but are likely to be in a more dynamic state. We postulate that SMAD3 and EZH2 regulate the accessibility of heterochromatin at other lineage specific genes in a highly conserved manner.

We have shown that pre-treatment of kidney organoids with the selective EZH2 inhibitor, GSK343, prior to TGFβ1 results in the attenuation of fibrotic gene expression and this correlates with the inhibition of TGFβ1-induced changes in chromatin accessibility. While GSK343 did not significantly change the identity of clusters, several SMAD target genes were clearly downregulated, likely reflecting the pleiotropic nature (SMAD-dependent and non-SMAD dependent) of TGFβ1 signalling. In many contexts, inhibition of EZH2 results in the transcriptional inactivation of the TGFβ1 signalling pathway^10,39,46^. Furthermore, inhibition of HDACs, which permit repression of gene expression, also results in dampening of the TGFβ1 transcriptional response^38,47–49^. Previous studies in our lab have shown that double knockdown of both SMAD3 and EZH2 in epithelial cells undergoing trans-differentiation in response to TGFβ results in the complete loss of fibrotic gene expression and retention of the epithelial junction marker, CDH1^10^. This would suggest that SMAD3 appears to require the recognition of compacted chromatin to bind and facilitate chromatin remodelling prior to establishing its transcriptional response. This observation is in line with those of others who have shown that SMAD proteins can bind to inactive chromatin and recruit chromatin remodellers in the absence of a pioneer transcription factor^50^.

Our study remains somewhat inconclusive pertaining to how SMAD3 and EZH2 interact at the chromatin level, however, we can speculate that EZH2 activity is required for this interaction to take place, in turn suggesting that genomic co-occupancy is a prerequisite; although, this might yet involve additional “linker” proteins as part of a wider complex. In the absence of EZH2, it is possible that phosphorylated SMAD3 persists in a non-complexed form. We suggest that SMAD3 increases chromatin accessibility in one of three ways. GSK343 binds inside the SET domain overlapping the SAM binding site^30,51^ therefore inhibiting interactions within the binding pocket. Therefore, a first possibility is that SMAD3 recognises and interacts with EZH2, perhaps at the catalytic SET domain, or alternatively at another site on the EZH2 molecule. A second possibility is that SMAD3 indirectly interacts with EZH2 through miRNAs or a related mechanism. A third potential mechanism by which SMAD3 may recognise the H3K27me3 mark is through interaction with histone readers (Supplementary Fig. 12). One candidate is the EED subunit of PRC2, which has H3K27me3 binding ability via its WD40 domain. This binding enhances PRC2 activity through a positive feedback loop that allows PRC2-mediated compaction to spread to adjacent nucleosomes^52^. Additional candidates include the Polycomb-like proteins PHF1 and PHF19, which we detected in EZH2 affinity purification experiments;^10,12^ Both proteins can enhance PRC2 catalytic activity^53,54^. However, SMAD3 has been shown to bind and form complexes with many histone readers, including those containing plant homeodomains, chromo- or bromodomains that bind and recognise histone modifications to promote SMAD-mediated transcriptional activity, so the range of candidates that SMAD3 may interact with is wide^55,56^.

To the best of our knowledge, this is the first time that scRNAseq and scATACseq datasets have been integrated to examine TGFβ1-induced changes in chromatin accessibility within fibroblast populations undergoing differentiation to myofibroblasts. Transcriptional regulation by SMAD complexes requires the remodelling of chromatin and this may be facilitated through the cooperation of SMAD3 with other transcription factor complexes. For example, SMAD3 and AP1 binding motifs were highly enriched in the activated myofibroblast cluster. In addition, we identified high AP1 regulon activity in MFib1 in response to TGFβ1, as well as increased regulon activity for ETS family members and other fibrosis-associated transcription factors (CREB3L1, NUAK1, RARG); all of which are increased in the fibrotic kidney^57–59^. In mammary epithelial cells undergoing trans-differentiation, SMAD3 has been thought to interact with AP1 to facilitate increased chromatin accessibility^35^ at enhancer regions^60^. AP1 is important for the selection and increased accessibility of enhancers in different cell types through the recruitment of SWI/SNF^61^. Furthermore, previous studies have established that SMARCA4 is required for the transcription of many TGFβ target genes^50,62^. We speculate that SMAD3 may recruit and/or cooperate with AP1 to identify specific loci to facilitate enhancer opening; this cooperation leads to the recruitment of chromatin remodelling complexes such as SWI/SNF, which displaces nucleosomes to facilitate chromatin accessibility and antagonism of the polycomb repressive complex. The displacement of PRC2/EZH2 complex and removal of the H3K27me3 mark requires the recruitment of JmjC-domain containing histone demethylases UTX and JMJD3 which demethylate tri-methylated H3K27 allowing for increased chromatin accessibility^63^. SMAD3 is known to recruit and physically interact with JMJD3/UTX during differentiation^64^ and cellular reprogramming^65^ and along with the ATP-dependent chromatin remodeller, CHD8, they establish an accessible chromatin landscape and activation of genes required for cell fate specification^66,67^. It is possible that SMAD3 simultaneously recruits histone demethylases like JMJD3 to facilitate increased accessibility. This provides the clearest picture to date of a TGFβ1-induced fibrotic fingerprint, critical to myofibroblast activation.

Formation of concentration gradients (condensates) of transcriptional machinery at regulatory regions may determine what genes should be switched ‘on’ or ‘off’^68–71^. Few pioneer transcription factors orchestrate condensate formation and can simultaneously interact with many different co-activators in a disordered fashion through their activating domain^68^. Heterochromatin has been shown to form dynamic liquid-like condensates consistent with those of activation condensates^72^. While activation and heterochromatin condensates do not overlap^72^, we surmise that changes in transcription factor and co-factor concentration gradients may allow for a dynamic switch between the two phases. This model of gene regulation may point to a mechanism by which SMAD3, through rapid chromatin interactions, can dynamically induce the remodelling of chromatin and interact simultaneously with various co-factors (e.g. HATs, co-activators, co-repressors, PRC2, and/or HDACs) to activate and repress transcription.

In summary, the work presented in this manuscript has generated a better understanding of the epigenetic mechanisms by which the TGFβ signalling pathway governs cell fate during differentiation in kidney development and disease. We propose that that the enzymatic function of the polycomb repressive complex is necessary for TGFβ1-induced increase in chromatin accessibility and its subsequent gene regulatory functions. Before we can exploit this mechanism for therapeutic benefit, further evaluation is necessary to determine how the cooperation between TGFβ and chromatin facilitates the regulation of genes necessary for fate specification in both physiological and pathological contexts.

## Methods

### Human iPSC maintenance

All experiments were performed in the human iPSC line HPSI1213i-babk_2 purchased from the ECACC. iPSCs were maintained on Vitronectin XF (StemCell Technologies, cat. no. 07180) coated plates in Essential E8 Flex (ThermoFisher Scientific, cat. no. A2858501) at 37 °C/5% CO_2_. Cell passaging was performed using ReLeSR™ (StemCell Technologies, cat. no. 05872) according to the methods described by the manufacturer.

### Kidney organoid generation

iPSCs at passages 32–38 were differentiated using an adapted protocol from^13^. Briefly, 15,100 cells per cm^2^ were seeded prior to differentiation. On the day of differentiation (Day 0), medium was switched to STEMdiff APEL2 medium (StemCell Technologies, cat. no. 05275) supplemented with 8 µM CHIR99021 (Sigma-Aldrich, cat. no. SML1046), 5% (v/v) PFHM-II protein free hybridoma medium (ThermoFisher Scientific, cat. no. 12040077), and 1% (v/v) antibiotic-antimycotic (100X) (AA) (ThermoFisher Scientific, cat. no. 15240062). On Day 3, medium was switched to APEL2 supplemented with 200 ng ml^−1^ human FGF9 (StemCell Technologies, cat. no. 78161.1), 1 µg ml^−1^ heparin (Sigma-Aldrich, cat. no. H4784), 5% (v/v) PFHM-II, and 1% (v/v) AA. Medium was changed every 48 h. On day 7, progenitor cells were cultured in 5 µM CHIR99021 for 1 h prior to pelleting at 300 × g for 3 minutes. Pellets were transferred onto 0.4 µm pore PET transwell filters and cultured until day 12 in APEL2 supplemented with FGF9 and heparin. On day 12, growth factors were removed, and organoids were matured until day 24. Medium was replenished every 48 h over the course of differentiation. On Day 24, Organoids were treated with 10 ng/ml human recombinant TGFβ1 (Promokine) for 48 h with repeated exposure after 24 h. 0.1% (w/v) BSA served as vehicle control. Organoids from the same batch were pre-treated with or without 5 µM GSK343 (Sigma Aldrich, cat. no. SML0766) for 1 h prior to TGFβ1 treatments. DMSO served as vehicle control.

### Immunofluorescence of organoid sections

Organoids (Day 7 or Day 26) were fixed in 2% (v/v) paraformaldehyde and cryoprotected in a 10-30% (w/v) sucrose gradient. Organoids were snap frozen in 7.5/10% (w/v) gelatine/sucrose at −70 °C using an isopropanol bath. Organoids were cryosectioned into 10-20 µm sections using a Leica CM3050S cryostat set to −20 °C. Sections were warmed to RT and antigen retrieval was performed using 1% (w/v) SDS in DPBS for 10 minutes. Sections were blocked for 1 h at RT using blocking solution (5% (v/v) goat serum, 0.1% (v/v) triton X-100, DPBS). Sections were incubated in primary antibody at 4 °C overnight. For LTL, sections were blocked using a streptavidin-biotin blocking kit before incubation with LTL (5 µg ml^−1^; Vector Labs, cat no. B-1325-2). Following incubation, sections were washed with DPBS and incubated overnight at 4 °C with Hoechst33342 (1:1000), and the corresponding fluorescence-conjugated secondary antibody (ThermoFisher, cat. no. A-11001 and A-11011) diluted in 5% (v/v) goat serum (Sigma Aldrich, cat no. G9023). For LTL, sections were washed with DPBS, incubated with streptavidin Dylight 649 (Vector Labs, cat. no. SA-5649-1) for 20 min at RT, and Hoeschst33342 (1:500) (ThermoFisher Scientific, cat. no. H3570) for 10 min at RT. Imaging was performed on a Leica SP8 or Zeiss LSM800 confocal microscope using a 20X air or 40X oil objective. For alpha smooth muscle actin and periostin staining, exposure times and laser power were kept consistent between all conditions. Antibodies and dilutions were: CDH1 (1:300; 610181, BD Bioscience), Laminin (1:300; L9393, Sigma), ZO-1 (1:300; 61-7300, Invitrogen), MEIS1/2/3 (1:100; sc-101850, Santa Cruz), WT1 (1:200; sc-393498, Santa Cruz), SIX2 (1:300, 11562-1-AP, Invitrogen), periostin (POSTN) (1:300; PA534641, Invitrogen), PDGFRA (1:300; PA516571, Invitrogen), PDGFRB (1:300; 3169, Cell Signalling), and Alpha smooth muscle actin (αSMA) (1:300; A5228, Sigma). Brightness and contrast adjustments were made using Fiji/ImageJ.

### Immunofluorescence of adherent cells

iPSCs at passages 32 to 38 were differentiated for 7 days on 8-well Ibidi µ-slides using an adapted protocol from^13^. Cells differentiated for 0, 3, and 7 days were fixed in 4% (v/v) paraformaldehyde for 10 min at RT and blocked for 1 h at RT using blocking solution containing 5% (v/v) goat or donkey serum. Cells were incubated in primary antibody at 4 °C overnight. Following incubation, cells were washed with DPBS and incubated overnight at 4 °C with Hoechst33342, and their corresponding fluorescence-conjugated secondary antibody (1:200-500). Images were acquired using a Leica SP8 confocal microscope. Antibodies and dilutions used were OCT4 (1:300; sc-5279, Santa Cruz), Brachyury (1:100; AF2085, R&D), HOXD11 (1:200; SAB1403944, Sigma), PAX2 (1:200; 71-6000, Invitrogen).

### Dextran uptake assay

Organoids at day 26 were cultured with 10 µg ml^−1^ of 10,000 MW dextran Alexa647-conjugated (ThermoFisher, cat. no. D22914) for 24 h. Organoids were fixed and stained without permeabilization. Images were acquired using a Leica SP8 confocal microscope.

### Histology

Sections were stained with Mayer haematoxylin for 6 min, followed by washing in warm running tap water for 5 min. Sections were stained with 0.5% (v/v) eosin Y for 6 min and washed for 3 min in running distilled water. Sections were dehydrated using 95% and 100% ethanol. Sections were incubated in Xylene before mounting using DPX Mountant. For picrosirius red staining, sections were rehydrated in DPBS for 10 min and then post-fixed in 4% (v/v) PFA for 30 min at room temperature. Sections were stained using the picrosirius red stain kit (Polysciences,cat. no. 24901) as per the manufacturer’s instructions. Sections were dehydrated using 100% ethanol and incubated in xylene before mounting using DPX mountant. Sections were imaged using a 10X objective and a Canon EOS600D camera installed on a Nikon 80i transmission light microscope.

### Statistics and reproducibility

Results are representative of the similar observations and analyses made across multiple independent experiments and technical replicates. Independent experiments were classed as monolayer differentiations or organoids derived from separate passages and/or freezebacks. The number of replicates for each experiment is indicated in the legends of the corresponding figures. Organoids used in single-cell RNA and ATAC sequencing experiments were differentiated from a single well of hiPSCs and three organoids were pooled as part of a single independent experiment and dissociated for scRNAseq and scATACseq. ChIPseq experiments included cells from two independent experiments. Organoid characterisation was performed on 3–4 organoids from a minimum of 3 independent experiments. Immunofluorescent analysis was performed on one organoid per condition, from three independent experiments (Figs. 3d-f and 4d-f). Histological analysis was performed on one organoid per condition, from four independent experiments (Supplementary Fig. 8). Wherever possible, commonly available tools and statistical methods were used. Fiji/ImageJ (version: 2.1.0/1.53q)^73^ was used for quantification of αSMA/DAPI and POSTN/DAPI areas, and quantification of picrosirius red staining. Statistical analysis was performed in GraphPad Prism (version: 8.3.0) and *P*-values were estimated by unpaired *t*-test.

### Transmission election microscopy

Organoids were fixed in 2% (v/v) PFA and 2.5% (v/v) glutaraldehyde in 0.1 M Sorensens phosphate buffer (0.133 M Na_2_HPO_4_, 0.133 M KH_2_PO_4_) overnight at 4 °C. Organoids were incubated in 1% (v/v) osmium tetroxide for 1 h at RT, followed by incubation in 1% (v/v) tannic acid for 1 h at RT. Organoids were dehydrated in 70, 90, and 100% ethanol. Organoids were incubated in a 50:50 mix of 100% ethanol: Agar 100 EPON epoxy resin (48.6% Agar 100 epoxy resin, 18.2% DDSA dodenyl succinic anhydride, 30.4% methyl nadic anhydride, 2.8% BDMA benzyldimethylamine) overnight on a rotator at RT. Organoids were then incubated in 100% Agar 100 EPON for 4 hours at 37 °C to evaporate any remaining ethanol. After incubation, organoids were polymerised in fresh EPON at 60 °C overnight. Ultrasections were imaged using a FEI Tecnai T12 transmission electron microscope at an accelerating voltage of 120 Kv. Images were analysed using ImageJ/Fiji. Semi-thin sections were stained with toluidine blue at 60 °C for 1 min and washed with ddH_2_O water. Coverslips were mounted using DPX and sections were imaged using a Nikon E80i transmission light microscope.

### Western blotting

iPSCs were differentiated for 7 days using an adapted protocol from^13^. Whole cell protein extracts were isolated and western blotting was performed using standard western blotting protocols using 8-12% polyacrylamide gels. Primary antibodies used were H3K27me3 (1:500; 39155, Active Motif), H3K4me3 (1:1000; ab12209, Abcam), EZH2 (1:5000; 5246, Cell Signalling), SMAD3 (1:2000; ab28379, Abcam), phosphorylated SMAD3 (1:2000; ab52903, Abcam). Beta-actin (1:20,000; A5316, Sigma) served as a loading control. Detection was performed using Advansta WesternBright ECL (Advansta, cat. no. K12045) and the Vilber Fusion XF Imager.

### ChIP-Seq

#### Chromatin shearing

iPSCs and differentiated progenitors used for ChIP-seq experiments were obtained from two independent experiments. iPSCs were grown in 100 mm plates and differentiated for 0 or 7 days using an adapted protocol from^13^. Chromatin from 5 × 10^7^ cells was prepared for shearing using the Covaris truChIP Shearing Kit (Covaris, cat. no. 520237) according to manufacturer’s protocol. Chromatin was sheared in a pre-cooled AFA microtube using a Covaris E220 Evolution AFA focused-ultrasonicator for 20 min at duty factor of 2%, 200 cycles per burst, and peak incident power of 140. Immunoprecipitation was performed with 3 µg Smad3 (Abcam, cat. no. ab28379) or 2.5 µg EZH2 (Cell Signalling, cat. no. 5246) antibodies as previously described^14^. Briefly, Protein G magnetic dynabeads (ThermoFisher, cat. no. 10003D) were pre-blocked in 1 x PBS with 0.5% BSA before incubation with antibodies on a rotator overnight at 4 °C. Sheared chromatin was diluted four times in dilution buffer (140 mM NaCl, 50 mM HEPES pH 8.0, 1 mM EDTA, 0.75% Triton X-100, 0.1% Na-deoxycholate, 1X protease inhibitor cocktail) and mixed with 30 µl of dynabead-antibody mix. Samples were incubated overnight by rotation at 4 °C. Beads were subsequently washed once with low salt buffer (20 mM Tris-HCl pH 8.0, 150 mM NaCl, 2 mM EDTA pH 8.0, 0.1% SDS), high salt buffer (20 mM Tris-HCl pH 8.0, 500 mM NaCl, 2 mM EDTA pH 8.0, 0.1% SDS), LiCl buffer (10 mM Tris-HCl pH 8.0, 1 mM EDTA pH 8.0, 250 nM LiCl, 1% IGEPAL), and twice with TE buffer containing 50 mM NaCl (10 mM Tris-HCl pH 8.0, 1 mM EDTA pH 8.0, 50 mM NaCl). Samples were eluted in 100 µl elution buffer (50 mM Tris-HCl pH 8.0, 10 mM EDTA pH 8.0, 1% SDS) at 65 °C for 45 min with vortexing every 5 min. Samples were centrifuged at 16,000 × *g* for 1 min at RT. Whole cell extract (WCE) to be used for input was diluted three times with elution buffer. Crosslinking was reversed in all samples by incubating at 65 °C overnight. 100 µl TE buffer was added to each sample to dilute SDS in elution buffer. Reverse-crosslinked DNA was treated with RNase A and Proteinase K for 2 h at 37 °C and 55 °C, respectively. Chipped DNA was purified using the QIAquick PCR purification kit (Qiagen, cat. no. 28104) and eluted in 70 µl of elution buffer. ChIP libraries were prepared using the Illumina TruSeq ChIP sample preparation kit (Illumina, cat. no. IP-202-1012). Single-end 1 × 75 sequencing was carried out on an Illumina NextSeq 550 platform, using dual-index Illumina adapters.

### Single-cell RNA sequencing

Organoids used in single-cell RNA sequencing experiments were differentiated from a single well of passage 35 hiPSCs. On day 24, three organoids were pooled as part of a single independent experiment and dissociated for scRNAseq. Organoids were dissociated into single-cell suspension using the cold-active protease method adapted from^74^. Organoids were dissociated in dissociation buffer (10 mg ml^-1^ *Bacillus lichenformis* (Sigma-Aldrich, cat. no. P5380), 125 U ml^−1^ DNase I (ThermoFisher, cat. no. 90083), 5 mM CaCl_2_ in DPBS) by gentle trituration for 15 min on ice. Cells were collected using 40 µm and 70 µm MACS SmartStrainers and centrifuged at 300 xg for 5 min. Pellets were resuspended in 1X PBS with 2% BSA and filtered using a 40 µm Flowmi cell strainer (Sigma Aldrich, cat. no. BAH136800040). Cell concentrations and viability were assessed using trypan blue staining. 10,000 single cells were loaded onto the 10X Chromium chip using the Single Cell 3′ Reagent Kit (version: 3.1) as per manufacturer’s protocol. Following library preparation and quantitation, libraries were sequenced on the Illumina NextSeq 550 platform.

### Single-cell ATAC sequencing

Organoids used in single-cell ATAC sequencing experiments were differentiated from a single well of passage 34 hiPSCs. On day 24, three organoids were pooled as part of a single independent experiment and dissociated for scATACseq as previously described. Cells were lysed for 5 min in 100 µl chilled 0.1X lysis buffer (10 mM Tris-HCl (pH 7.4), 10 mM NaCl, 3 mM MgCl_2_, 1% BSA, 0.01% Tween-20, 0.01% IGEPAL CA-630, 0.001% Digitonin). Nuclei concentration and viability were determined using ethidium homodimer-1 (2 mM). scATACseq libraries were generated using 10X Genomics Chromium ATAC library and Gel Bead Kit (version: 1.1) according to the manufacturer’s protocol. Libraries were sequenced using an Illumina NovaSeq 6000 platform.

### ChIP-seq data analysis

Illumina BaseSpace was used to align sequences to hg19 genome. Peak calling was performed on merged replicates using EaSeq (version: 1.111)^75^ using the adaptive local thresholding method and default settings. Peaks overlapping blacklisted features as defined by the ENCODE project^76^ were removed. Data visualisations were performed in EaSeq. Chromatin state segmentation was performed for iPSCs using ChromHMM from ENCODE on UCSC Genome Browser (GRCH37/hg19)^77,78^. Chromatin state segmentation was performed for nephron progenitors using the 25-state chromatin model of the human foetal kidney (17 gestation weeks) epigenome (EID: E086; Donor/Sample ID: H-22676) from^79^. For motif analysis, peak sequences were generated using EaSeq. Sequences were extracted from fasta files of the genome assembly ‘hg19’ loaded from UCSC^80,81^. Homer (version: 4.11)^82^ was used to perform comprehensive motif analysis using the default settings^83^. Potential enhancers were identified using human embryonic stem cell and foetal kidney data on Enhancer Atlas (version: 1.0)^84^ and GeneHancer (version: 4.8)^85^. BigWig tracks for all samples were generated using the bamCoverage tool (Bin size = 25 bp) on the public server at usegalaxy.org^86^. BigWig tracks were visualised using the UCSC Genome Browser^87^.

### scRNAseq analysis

Cell Ranger (version: 3.1.0) was used to demultiplex, align, and generate single cell feature counts. scDblFinder was used to remove suspected doublets. Seurat (version: 3.0)^88^ was used to filter low-quality cells (containing unique genes or UMIs >2 standard deviations above the median for all samples or containing >25% mitochondrial reads). After QC, 15,583 high quality cells were obtained (Control: 4057; TGFβ1: 4119, GSK343: 3584; GSK343 + TGFβ1: 3823). scRNAseq datasets were normalised using SCTransform based normalisation^88^, regressing out % mitochondrial reads and the difference in G2M and S cell cycle score as calculated by Seurat. Next, dimensional reduction by principal component analysis was performed based on the top 2000 variable features as calculated by SCTransform. Sixty principal components were selected to construct a K-nearest neighbour graph. Using a resolution of 0.5, cells were clustered and classified into 17 cell types by sub-clustering and combining detected clusters as needed. To visualise these clusters, uniform manifold approximation and projection (UMAP) was generated using the same principal components used to construct the K-nearest neighbour graph. Cell type assignment was performed based on positive and negative marker genes identified in other organoid datasets. Gene ontology was performed using Panther (version: 17.0)^89,90^.

### Cell cycle analysis and core matrisome scoring

Cell cycle phase scores were calculated using Seurat. The expression of core matrisome and TGFβ genes previously described^91,92^ were summarised based on normalised gene expression data using the same method used for cell cycle analysis.

### Trajectory analysis

Heat diffusion for affinity-based transition embedding (PHATE) was used to identify temporal cell trajectories and map cell fate lineages for each of the clusters^93^. Trajectories were visualised using the Cerebro software application (version: 1.2)^94^. Pseudotime trajectories were visualised using the using BBrowser software (version: 2.10) (Bioturing, https://bioturing.com).

### Differential expression testing

Differential expression testing between stromal clusters was performed using the Wilcoxon rank sum test. Differentially expressed gene networks were analysed and visualised using Cytoscape (version: 3.8.1)^95^.

### Protein-protein interaction and gene ontology analysis

Protein-protein interaction networks were obtained using the public STRING database using a confidence score of 0.4 or greater for interactions. Functional gene ontology enrichment was obtained using the STRING enrichment app (version 11.5)^96^.

### scATACseq analysis

Cell Ranger ATAC (version: 1.2.0) was used to for demultiplexing and alignment. All four conditions were aggregated together. scATACseq datasets were pre-processed using Signac (version: 1.1)^97^. To perform quality control, cells with peak region fragments <3000 or >20,000, % reads in peaks < 15, blacklist ratio > 0.05, nucleosome signal > 4, and transcription start site (TSS) enrichment < 2 were removed.

To visualise the data, uniform manifold approximation and projection (UMAP) was generated using the same reduction method used to construct the K-nearest neighbour graph. To visualise the predicted activity of canonical marker genes, a gene activity matrix was generated, and log normalised using fragment counts from extracted coordinates located up to 2 kb upstream of gene promoters and within the gene bodies. Cell type annotation was performed using the label transfer feature of Seurat to transfer cell type identities from the scRNAseq data to the scATACseq data. First, anchors were identified from the gene activity matrix to transfer cell type cluster labels between data sets. Correlated patterns between gene activity and RNAseq were scored and used to predict annotation. Transferred labels were combined with clustering based on the previously computed SVD-TDIF matrix, dataset integration using Harmony^98^, and predicted gene activity of cluster marker genes identified from the RNAseq to form the final cell type assignments. In total, labels from 13 cell types were transferred from the scRNAseq data to the scATACseq data. The remaining 4 types of cells identified in the scRNAseq data were comprised mainly of proliferating populations of cell types already present and could not be identified as separate clusters in the scATACseq.

### Chromatin accessibility changes at regulatory regions

To obtain a global view and cluster-based changes in accessibility within regulatory regions, the number of fragments located within classes of regulatory regions (Transcription start sites, Enhancers, Promoters, and DNAse hypersensitive regions) as annotated by Cell Ranger ATAC in each cell were extracted and divided by the total number of fragments within each cell which passed filtering by Cell Ranger ATAC. These scores were then scored as metadata within the Seurat object and visualised using the ggplot2 package. For statistical testing, the ggpubr and rstatix packages were used to compare the means between treatment groups by unpaired t-test with post-hoc Bonferroni correction for multiple testing.

### Cis co-accessibility

The Cicero package within Signac was used to construct and predict *cis* co-accessibility connections^31^. Differential accessibility testing between stromal clusters was performed using logistic regression test using the total number of fragments as a latent variable with a min.pct of 0.25. Gene ontology was performed using Enrichr^99^.

### Motif analysis

DNA sequence motif analysis was performed in Signac. To find overrepresented motifs for differentially accessible peaks identified in Fib2 and MFib1, motif position frequency matrices were extracted from the JASPAR2020 database^100^. To find cell-type specific regulatory sequences, a hypergeometric test was performed to examine the possibility of observing the motif at the given loci. Motif activities per cell were computed using chromVAR in Signac^101^. To perform transcription factor footprinting analysis, the expected Tn5 insertion frequency was computed for each instance of the input motif in Signac.

### SCENIC

SCENIC^102^ was used to determine potential regulatory transcription factors through the VSN nextflow pipeline (v0.26.1)^103^ using default settings according to the developer vignette (https://github.com/aertslab/SCENIC). As SCENIC is a stochastic algorithm and results can differ each time it is run depending on the generated random seed, the full scenic pipeline was iterated 100 times, retaining only regulons present in over 80% of runs. SCOPE^104^ was used for the initial exploration of the results. Further analysis was performed in R.

### Reporting summary

Further information on research design is available in the Nature Portfolio Reporting Summary linked to this article.

## Supplementary information

Supplementary Information

Description of Additional Supplementary Data

Supplementary Data 1

Supplementary Data 4

Supplementary Data 7

Supplementary Data 9

Reporting Summary

## Data Availability

The datasets that support the findings of this study have been deposited at ArrayExpress with the accession codes: ChIP-seq data (Accession number: E-MTAB-10910), single-cell RNAseq data (Accession number: E-MTAB-11138) and single-cell ATAC-seq data (Accession number: E-MTAB-11139). Source data for ChIPseq graphs (Supplementary Fig. 1a, b), and statistical analysis of immunofluorescence and histological images (Figs. 3e, f, 4e, f and Supplementary Fig. 8b) are available in Supplementary Data 9. Source data for western blots are available in Supplementary Fig. 13.

## Code availability

All R code used in this paper has been deposited on GitHub and is available at: https://github.com/CiaranKennedyUCD/Kidney_Organoid_Paper_scRNA_ATAC_seq (doi: 10.5281/zenodo.7311441).
