# Supplementary material for: Single-cell multiomics reveals the complexity of TGFβ signalling to chromatin in iPSC-derived kidney organoids
Source: Commun Biol. 2022 Nov 27;5:1301. doi: 10.1038/s42003-022-04264-1 (PMC9701233; doi:10.1038/s42003-022-04264-1)
Supplement: Supplementary file 1 — Supplementary Information [file 42003_2022_4264_MOESM1_ESM.pdf]

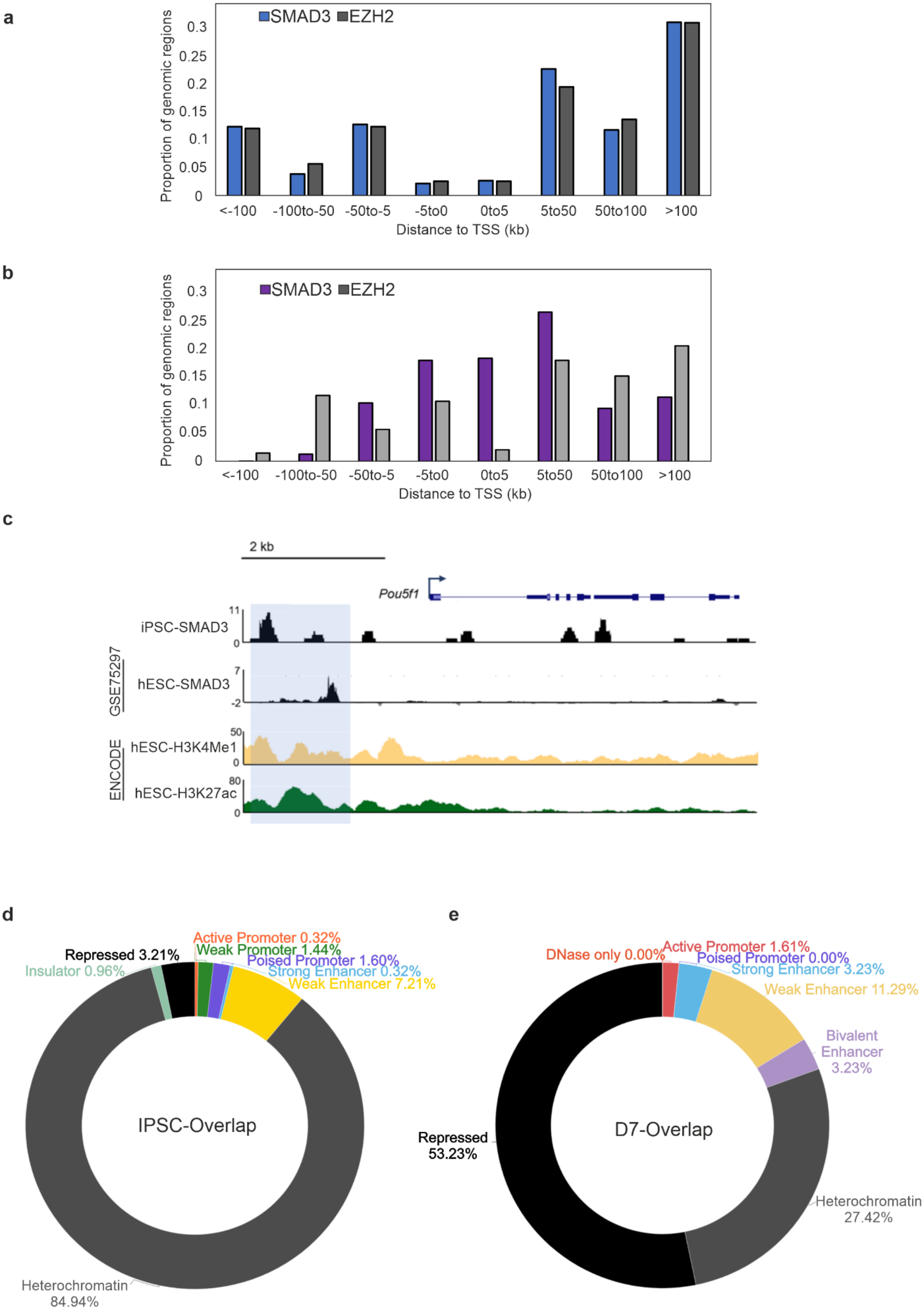
a
SMAD3 EZH2
Proportion of genomic regions
0.3
0.25
0.2
0.15
0.1
0.05
0
<-100
-100to-50
-50to-5
-5to0
0to5
5to50
50to100
>100
Distance to TSS (kb)
b
SMAD3 EZH2
Proportion of genomic regions
Distance to TSS (kb)
c
2 kb
Pou5f1
iPSC-SMAD3
hESC-SMAD3
GSE75297
hESC-H3K4Me1
hESC-H3K27ac
ENCODE
d
IPSC-Overlap
Active Promoter 0.32%
Weak Promoter 1.44%
Poised Promoter 1.60%
Strong Enhancer 0.32%
Weak Enhancer 7.21%
Repressed 3.21%
Insulator 0.96%
Heterochromatin 84.94%
e
D7-Overlap
DNase only 0.00%
Active Promoter 1.61%
Poised Promoter 0.00%
Strong Enhancer 3.23%
Weak Enhancer 11.29%
Bivalent Enhancer 3.23%
Repressed 53.23%
Heterochromatin 27.42%

Supplementary Figure 1: SMAD3 and EZH2 occupied sites in iPSCs and Day 7 intermediate mesoderm cells.

a. Histogram showing the proportion of genomic regions bound by SMAD3 (Blue; n=971) and EZH2 (grey; n=8528) with respect to the distance from a neighbouring TSS at 0-5, 5-50, and 50-100 kilobase (kb) intervals in iPSCs. **b.** Histogram showing the proportion of genomic regions bound by SMAD3 (Blue; n=971) and EZH2 (grey; n=8528) with respect to the distance from a neighbouring TSS at 0-5, 5-50, and 50-100 kilobase (kb) intervals. **c.** *Pou5f1* (OCT4) locus showing SMAD3 peaks from iPSCs and hESCs (Geo Accession number: GSE75297) as well as H3K4me1 and H3K27ac ChIP-seq peaks from the ENCODE project. Distribution of genomic features that were bound by both SMAD3 and EZH2 in **d.** iPSCs and **e.** nephron progenitors using ChromHMM annotation from Encode.

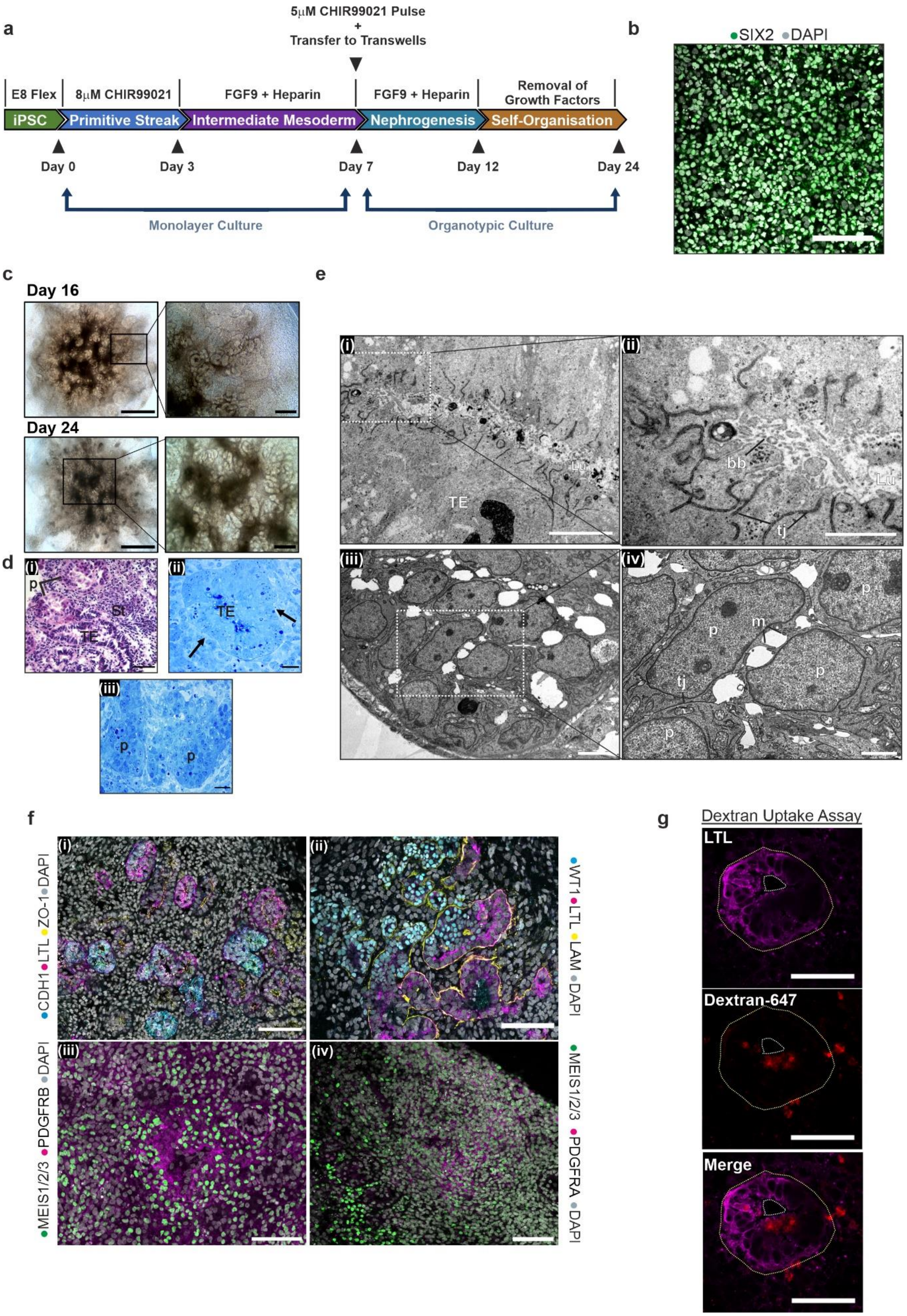

a
5μM CHIR99021 Pulse
+
Transfer to Transwells
E8 Flex
8μM CHIR99021
FGF9 + Heparin
FGF9 + Heparin
Removal of Growth Factors
iPSC
Primitive Streak
Intermediate Mesoderm
Nephrogenesis
Self-Organisation
Day 0
Day 3
Day 7
Day 12
Day 24
Monolayer Culture
Organotypic Culture
b
SIX2 DAPI
c
Day 16
Day 24
d
(i) p St TE
(ii) TE
(iii) p p
e
(i) Lu TE
(ii) bb Lu tj
(iii)
(iv) p m p tj p p
f
(i) CDH1 LTL ZO-1 DAPI
(ii) WT1 LTL LAM DAPI
(iii) MEIS1/2/3 PDGFRB DAPI
(iv) MEIS1/2/3 PDGFRA DAPI
g
Dextran Uptake Assay
LTL
Dextran-647
Merge

Supplementary Figure 2: Derivation and characterisation of kidney organoids

a. Kidney organoids were generated from human iPSCs using an adaptation of the protocol as described by Takasato et al. (2016). **b.** Cells were positive for SIX2 by immunofluorescence on Day 7 of differentiation. Scale: 100µm. **c.** Networks of tubular epithelial structures were apparent by day 16 and matured up to day 24. Magnification: 4X and 20X. Scale bar: 1mm and 250µm. **d. (i)** Haematoxylin and Eosin staining of kidney organoids showing developing podocyte-like structures (p) and tubular epithelial structures (TE) surrounded by interstitium (St). Toluidine blue staining of organoids showing the presence of **(ii)** tubular epithelia (black arrows) and developing podocyte-like structures. Magnification: **(i)** 20X. **(ii)** 60X. **(iii)** 40X. Scale bar: 50µm. **e.** Organoids contained appropriate **(i)** tubular epithelial structure with presence of brush-border (bb), tight junctions (tj) and lumen (Lu) and developing podocytes (p) containing tight junctions and microvilli (m). Magnification: **(i)** 4200X, **(ii)** 11500X, **(iii)** 2550X**, (iv)** 6000X**.** Scale bar: **(i)** 5µm, **(ii)** 1µm, **(iii)** 5µm, **(iv)** 2µm. **f.** Organoids demonstrated **(i)** a spatially organised segmented structure consisting of proximal (LTL/ZO-1^{+ve}) and distal (CDH1/ZO-1^{+ve}) tubular epithelial cells, (**ii**) immature podocytes (WT1^{+ve}), and **(iii)** interstitial cells (MEIS1/2/3^{+ve}, PDGFRA^{+ve}, PDGFRB^{+ve}). Basement membranes were positive for laminin (Lam) (**ii**). Scale bar: **(i)** 200µm**, (ii-iv)** 100µm. **g.** Organoids showed functionality by the uptake of fluorescently labelled 10 kDa dextran. Scale bar: 50µm. Images are representative of a minimum of three independent experiments.

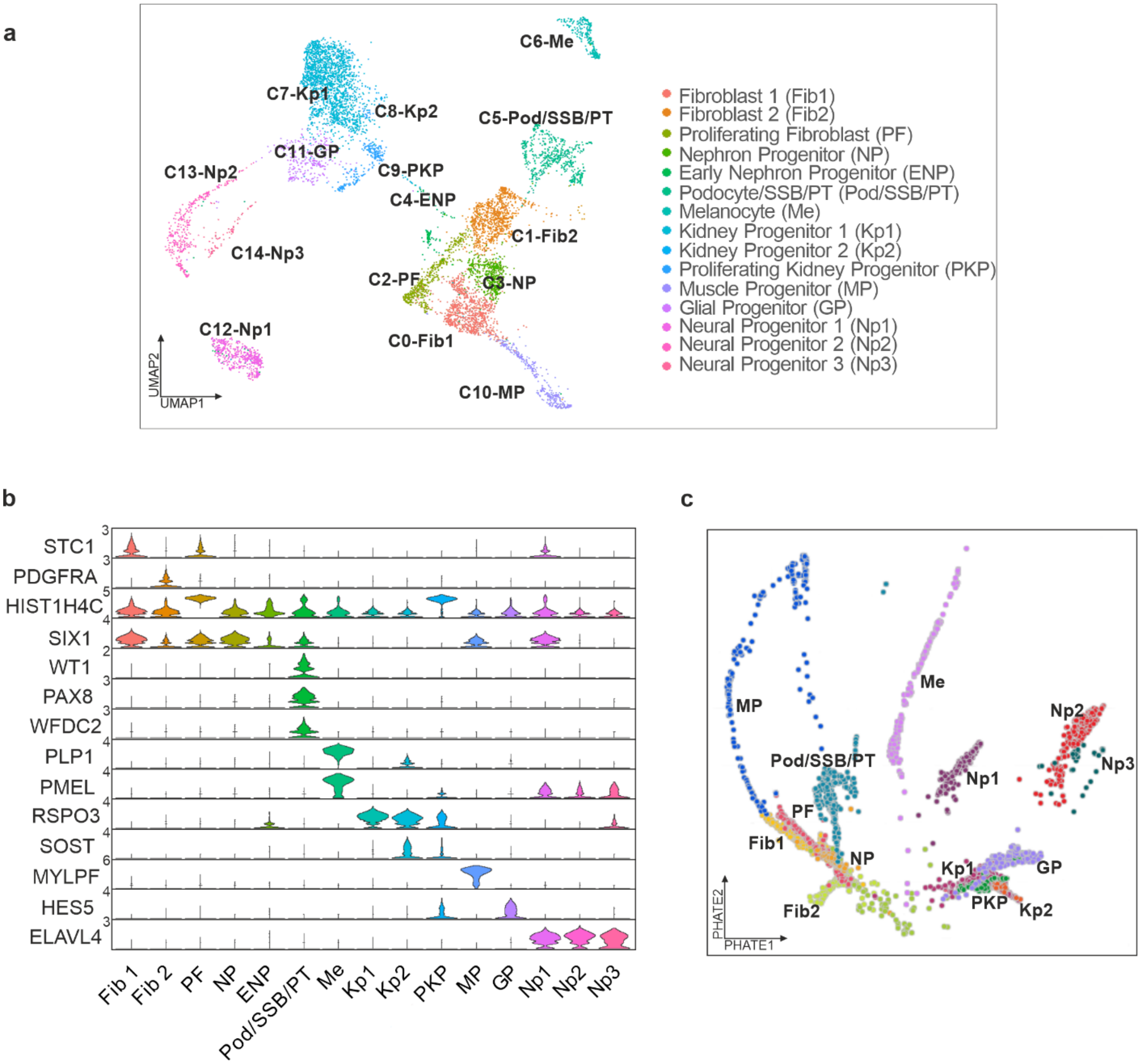

Supplementary Figure 3. Single cell RNA-seq characterisation of iPSC-derived kidney organoids

a. UMAP projection of 4,119 single cells revealing 15 distinct cluster in iPSC-derived kidney organoids, including distinct populations of fibroblasts, interstitial cells, and podocytes. SSB: S-Shaped Body; PT: Proximal Tubule. **b.** Marker genes used to annotate individual cell clusters. **c.** Cell Phate Map illustrating fate trajectories of each cell type within the organoid.

Supplementary Data 2: Gene ontology analysis (Panther) of top 15 differentially expressed genes per cluster iPSC-derived kidney organoids. SSB = S-shaped body, PT= proximal tubule.

Cluster	GO summary for 15 differentially expressed genes
Cluster 0<br>***Fib 1***	*Striated muscle tissue development (GO:0014706), Actin-myosin filament sliding (GO:0033275), Muscle filament sliding (GO:0030049), Muscle organ development (GO:0007517), epithelial cell differentiation (GO:0007517)*
Cluster 1<br>***Fib 2***	*Collagen fibril organisation (GO:0030199), Protein complex subunit organisation (GO:0071822), Regulation of epithelial to mesenchymal transition (GO:0010717), Extracellular matrix organisation (GO:0030198), Muscle contraction (GO:0006936)*
Cluster 2<br>***Proliferating fibroblasts***	*Sister chromatid segregation (GO:0000819), Mitotic nuclear division (GO:0140014), Chromosome condensation (GO:0030261), Mitotic sister chromatin segregation (GO:0007076), Establishment of mitotic spindle localisation (GO:0040001)*
Cluster 3<br>***Nephron Progenitor***	*Regulation of morphogenesis of a branching structure (GO:0001763), Regulation of morphogenesis of an epithelium (GO:0002009), Regulation of animal organ morphogenesis (GO:0009887), Positive regulation of cell proliferation (GO:00082884), histone-serine phosphorylation (GO:0035404)*
Cluster 4<br>***Early Nephron Progenitor***	*RNA splicing (GO:0008380), RNA processing (GO:0006396), RNA splicing via transesterification reactions (GO:0000377), mRNA splicing via spliceosome (GO:0000398), mRNA processing (GO0006397)*
**Cluster 5**<br>***Podocyte/SSB/PT***	*Mesenchymal to epithelial transition (GO:0060231), Renal system development (GO:0001822), Branching involved in ureteric bud morphogenesis (GO:0001658), Metanephros development (GO:0001656)*
**Cluster 6**<br>***Melanocyte***	*Autonomic nervous system development (GO:0048483), Positive regulation of cell junction assembly (GO:1901890), Divalent inorganic cation homeostasis (GO:0072507), Metal ion homeostasis (GO:0055065), Receptor metabolic process (GO:0043112)*
**Cluster 7**<br>***Kidney Progenitor 1***	*Regulation of Wnt signalling pathway (GO:0030111), Regulation of planar cell polarity pathway (GO:2000095), Positive regulation of gene expression (GO:0010628), Regulation of transcription from RNA Polymerase II (GO:0006357), Transmembrane receptor protein tyrosine phosphatase signalling pathway (GO:0007185)*

**Cluster 8**<br>***Kidney Progenitor 2***	*Vascular smooth muscle cell differentiation (GO:0035886), negative regulation of glial cell proliferation (GO:0060253), Negative regulation of oligodendrocyte differentiation (GO:0048715), Vascular smooth muscle cell differentiation (GO:0097084), Regulation of intrinsic apoptotic signalling pathway by p53 (GO:1902253)*
**Cluster 9**<br>***Proliferating Kidney Progenitor***	*Chromosome condensation (GO:0030261), Mitotic sister chromatin segregation (GO:0007076), Regulation of cell cycle G2/M phase transition (GO:1902749), Mitotic chromosome condensation (GO:0007076), Regulation of protein localisation to the nucleus (GO:1900180)*
**Cluster 10**<br>***Muscle Progenitor***	*Muscle filament sliding (GO:0030049), Actin-myosin filament sliding (GO:0033275), Muscle contraction (GO:0006936), Striated muscle contraction (GO:0006941), Skeletal muscle contraction (GO:0003009)*
**Cluster 11**<br>***Glial Progenitors***	*Negative regulation of neuron differentiation (GO:0045665), Glial cell differentiation (GO:0010001), Negative regulation of neurogenesis (GO:0060768), Regulation of cysteine-type endopeptidase activity (GO:2000116), Eye development (GO:0001654)*
**Cluster 12**<br>***Neural Progenitor 1***	*Regulation of dephosphorylation (GO:0035303), Substantia nigra development (GO:0021762), Nervous system development (GO:0007399), Glomerular epithelial cell differentiation (GO:0072311), Membrane raft assembly (GO:0061318)*
**Cluster 13**<br>***Neural Progenitor 2***	*Regulation of microtubule depolymerisation (GO:0031114), Neuron migration (GO:0001764), Generation of neurons (GO:0048699), Positive regulation of axon guidance (GO:1902669), Regulation of protein depolymerisation (GO:0048666)*
**Cluster 14**<br>***Neural Progenitor 3***	*Microtubule depolymerisation (GO:0007019), Central nervous system development (GO:0007417), Regulation of cytoskeleton organisation (GO:0051493), Neuron development (GO:0007399), Nervous system development (GO:0007399)*

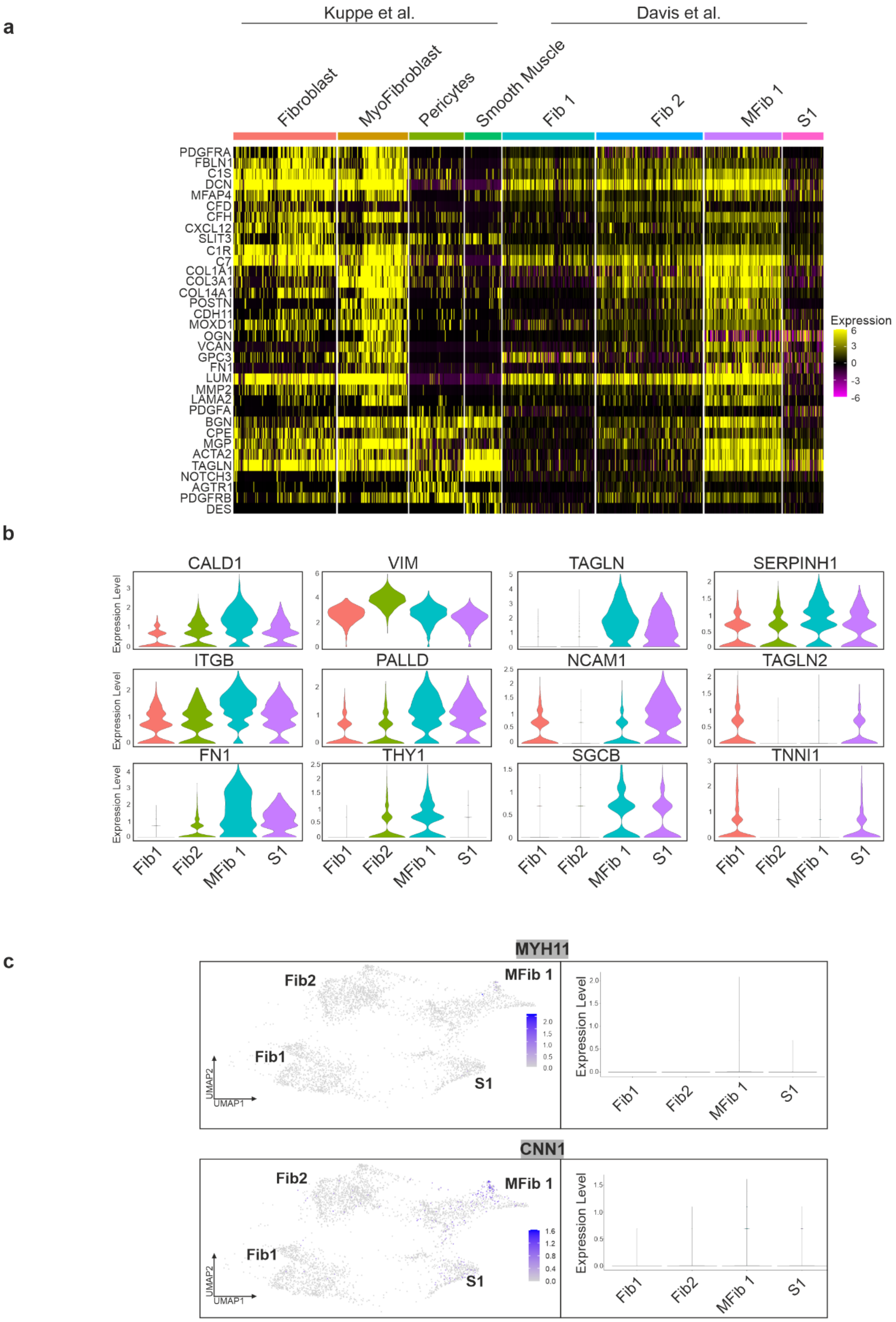
a
Kuppe et al.
Davis et al.
Fibroblast
MyoFibroblast
Pericytes
Smooth Muscle
Fib 1
Fib 2
MFib 1
S1
PDGFRA
FBLN1
C1S
DCN
MFAP4
CFD
CFH
CXCL12
SLIT3
C1R
C7
COL1A1
COL3A1
COL14A1
POSTN
CDH11
MOXD1
OGN
VCAN
GPC3
FN1
LUM
MMP2
LAMA2
PDGFA
BGN
CPE
MGP
ACTA2
TAGLN
NOTCH3
AGTR1
PDGFRB
DES
Expression
b
CALD1
VIM
TAGLN
SERPINH1
ITGB
PALLD
NCAM1
TAGLN2
FN1
THY1
SGCB
TNNI1
Expression Level
Fib1
Fib2
MFib 1
S1
c
MYH11
CNN1
Fib2
MFib 1
Fib1
S1
UMAP1
UMAP2
Expression Level

Supplementary Figure 4. Additional characterisation of stromal populations present within the untreated and TGFβ1-treated kidney organoids.

a. Comparison of Fib 1, Fib 2, MFib 1 and S1 with fibroblast, myofibroblast, pericyte and smooth muscle gene expression from previously published scRNAseq data from patients with renal fibrosis[23]. **b.** Violin plots of additional fibroblast and smooth muscle gene expression in Fib 1, Fib 2, MFib 1 and S1. **c.** UMAP plot of gene expression for smooth muscle genes MYH11 and CNN1 and their corresponding violin plots in Fib 1, Fib 2, MFib 1 and S1.

Supplementary Data 3: Gene ontology analysis (Panther) of top 15 differentially upregulated genes in MFib 1 and S1 in response to TGFβ1.

Cluster	GO Summary for 15 differentially expressed genes
Cluster 2 ***MFib 1***	*Collagen fibril organisation (GO:0030199), Protein complex subunit organisation (GO:0071822), Regulation of epithelial to mesenchymal transition (GO:0010717), Extracellular matrix organisation (GO:0030198), Muscle contraction (GO:0006936)*
Cluster 3 ***S1***	*Embryonic organ development (GO:0048568), Regulation of cytoskeletal organisation (GO:0051493), Negative regulation of platelet-derived growth factor receptor-beta signalling pathway (GO:2000587), Regulation of phosphatidylinositol biosynthetic process (GO:0010511), Left/Right pattern formation (GO:0060972)*

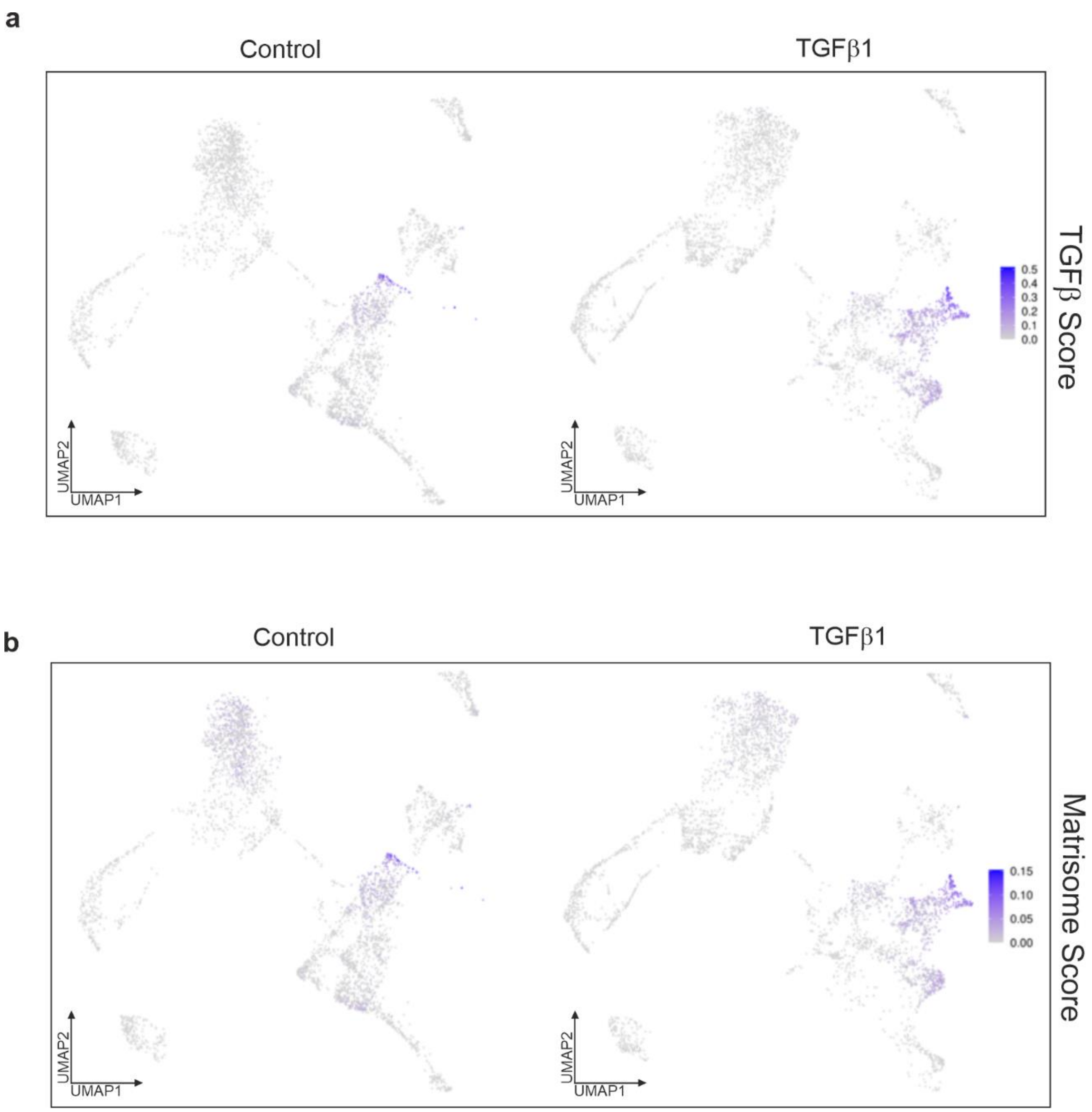

Supplementary Figure 5: Single cell RNAseq UMAP projections of "matrisome" and "TGFβ-regulated" genes in iPSC-derived kidney organoids treated with TGFβ1.

a. Cells within the control and TGFβ1-treated organoids were scored for TGFβ target genes. An increase in TGFβ associated genes was seen in clusters MFib 1 and S1. **b.** Cells within the control and TGFβ1-treated organoids were scored for core matrisome genes and these genes were also increased in MFib 1 and S1.

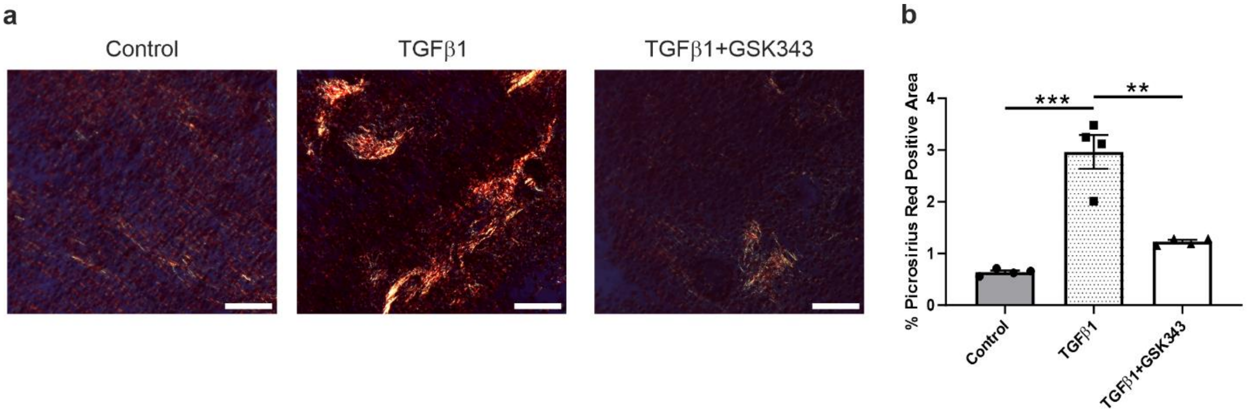

Supplementary Figure 6. Picrosirius red staining of untreated, TGFβ1, and TGFβ1+GSK343 treated organoids.

a. Representative collagen staining with Picrosirius red stain in untreated kidney organoids and organoids treated with TGFβ1 or TGFβ1+GSK343. Scale bar: 100µm. **b**. Percentage of positively stained picrosirius red area. Bar graph shows semi-quantitative analysis for picrosirius red stained sections, taken from one organoid per condition, from four independent experiments. Data are presented as the mean ± SEM. **$P < 0.0004$ (Control versus TGFβ1), ***$P < 0.0019$ (TGFβ1 versus TGFβ1+GSK343).

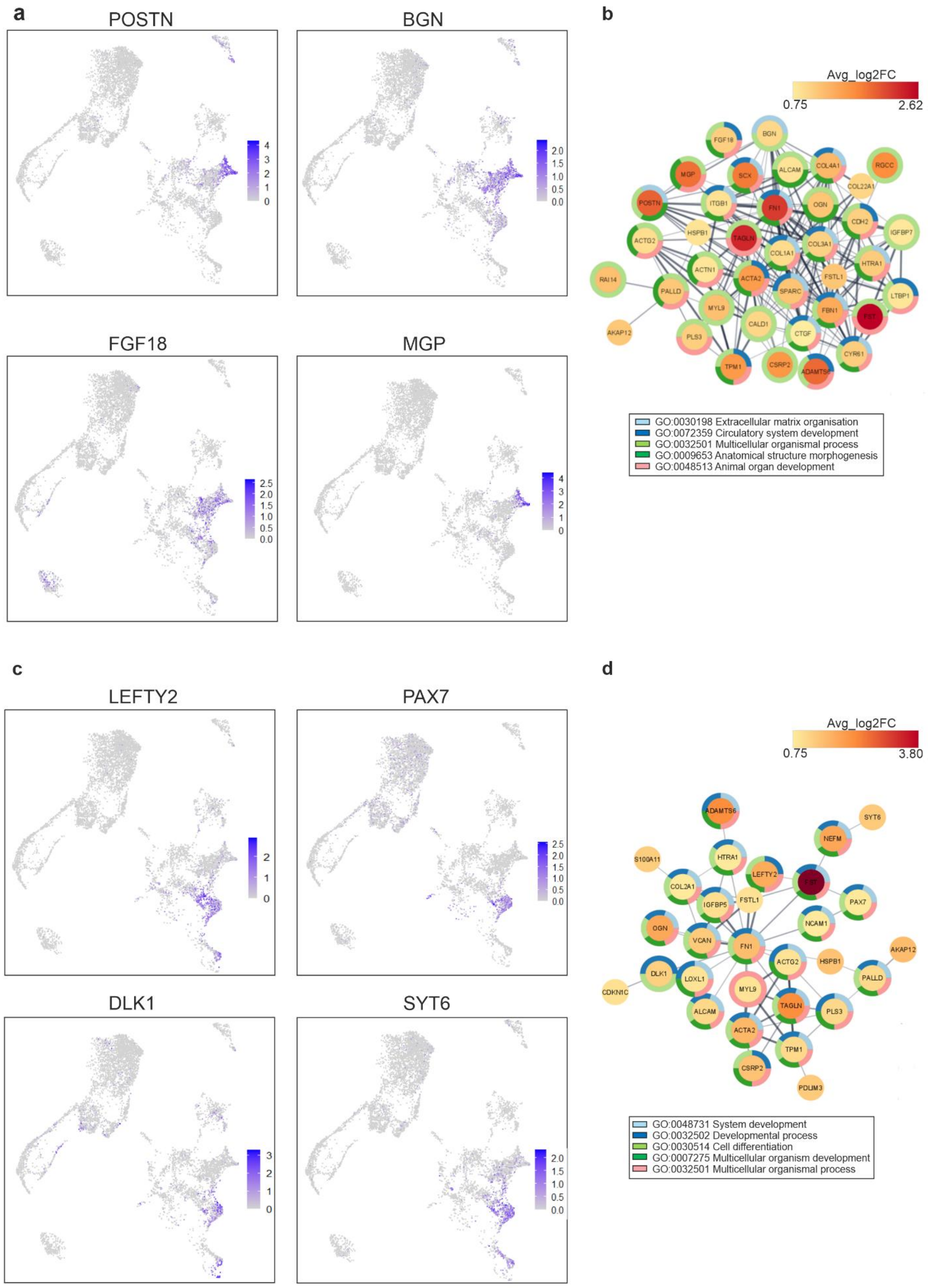
a
POSTN
BGN
FGF18
MGP
b
Avg_log2FC
0.75
2.62
GO:0030198 Extracellular matrix organisation
GO:0072359 Circulatory system development
GO:0032501 Multicellular organismal process
GO:0009653 Anatomical structure morphogenesis
GO:0048513 Animal organ development
c
LEFTY2
PAX7
DLK1
SYT6
d
Avg_log2FC
0.75
3.80
GO:0048731 System development
GO:0032502 Developmental process
GO:0030514 Cell differentiation
GO:0007275 Multicellular organism development
GO:0032501 Multicellular organismal process

Supplementary Figure 7. Myofibroblast associated genes are enriched in MFib 1 and S1. **a.** UMAP plots of genes that are upregulated in MFib 1 compared to Fib 1, 2 and S1 including the expression of P*OSTN, BGN, FGF18,* and *MGP*. **b.** Protein-protein interactions and gene ontology (biological process) analysis were generated using STRING in Cytoscape for the top 50 differentially upregulated genes in MFib 1. Nodes are coloured based on average log2 fold change (Avg_log2FC) and line thickness indicates strength of data supporting the interaction. Genes without identified interactions are not shown. The top 5 enriched GO terms for each gene are shown. **c.** UMAP plots of genes that are upregulated in S1 compared to Fib 1,2, and MFib 1 including the expression of *LEFTY2, PAX7, DLK1*, and *SYT6*. **d.** Protein-protein interactions for the top 50 differentially upregulated genes in S1.

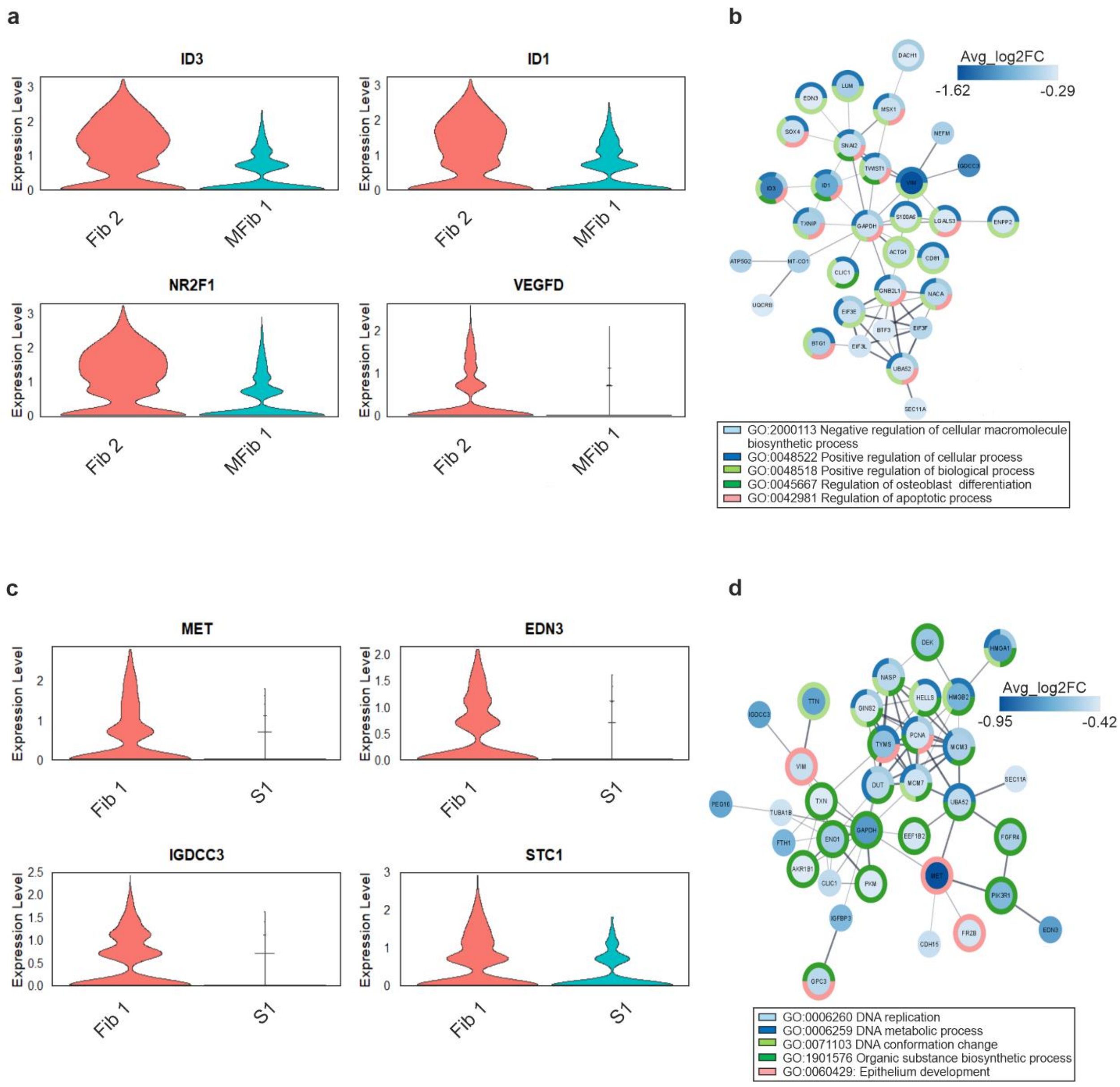

Supplementary Figure 8. Differentially downregulated genes in MFib 1 and S1 compared to parent populations.

a. Violin plots of differentially downregulated genes in MFib 1 compared to parent cluster Fib 2 including *ID3, ID1, NR2F1*, and *VEGFD*. **b.** Protein-protein interactions and gene ontology (biological process) analysis were generated using STRING in Cytoscape for the top 50 differentially downregulated genes in S1. Nodes are coloured based on average log2 fold change (Avg_log2FC) and line thickness indicates strength of data supporting the interaction. Genes without identified interactions are not shown. The top 5 enriched GO terms for each gene are shown. **c.** Violin plots of differentially downregulated genes in S1 compared to parent cluster Fib 2 including *MET, EDN3, IGDCC3,* and *STC1*. **d.** Protein-protein interactions and gene ontology (biological process) analysis for the top 50 differentially downregulated genes in S1.

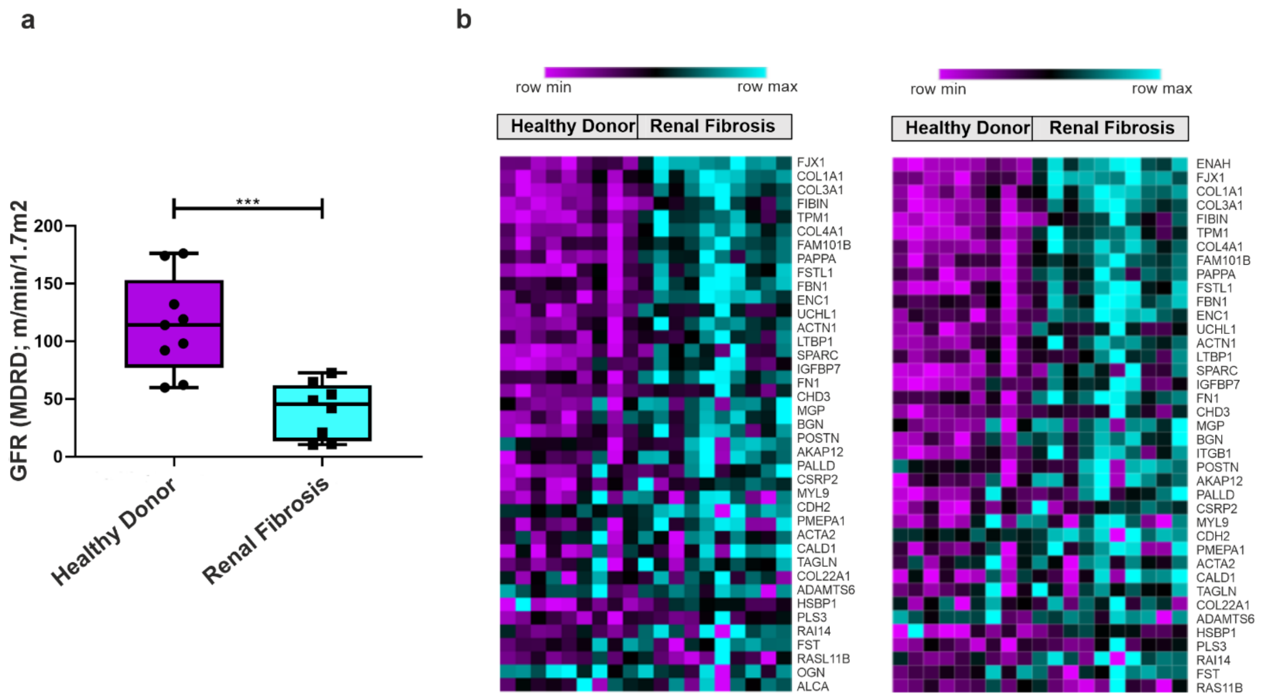

Supplementary Figure 9: Top upregulated genes in MFib and S1 compared to bulk RNAseq from patients with renal fibrosis.

A. Reduced GFR in renal fibrosis is characteristic of loss of renal function. ***P<0.0006, unpaired t-test. **B.** DEG genes from MFib 1 and Per 1 are like that observed in patients with kidney disease. Data was obtained from healthy donor (n=9) and kidney disease samples (n=10) from the European Renal cDNA bank Nephrotic Syndrome Tubulointerstitial dataset (www.nephroseq.org).

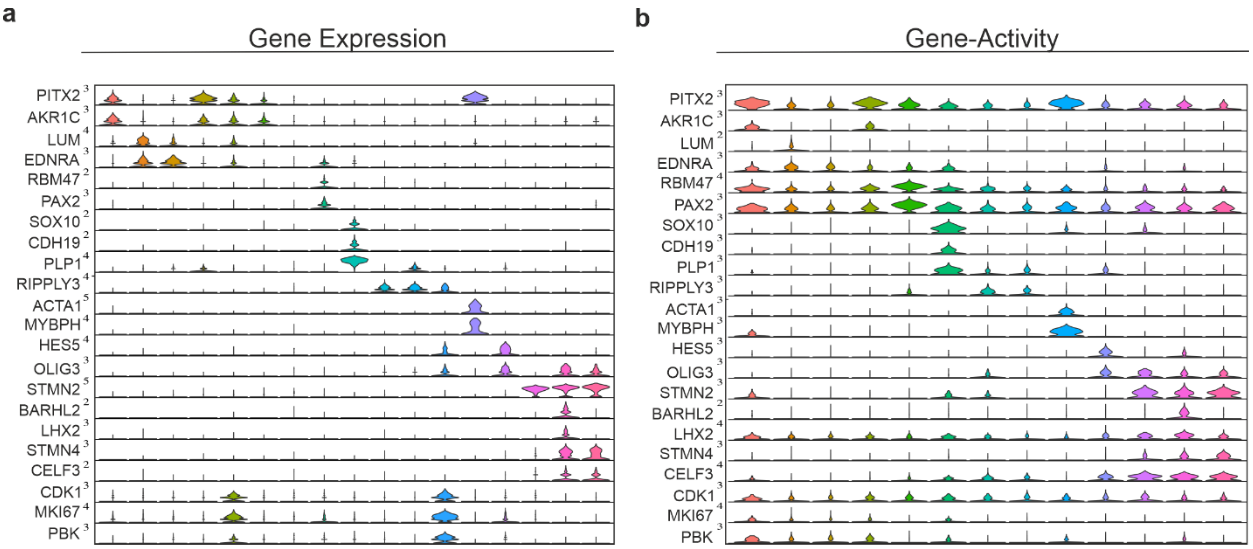

Supplementary Figure 10. Annotation of scATACseq clusters.

To interpret cluster annotation in the scATACseq dataset, cross-modality integration was performed, and labels were transferred. DotPlot visualisation of shared gene expression and gene-activity patterns for key marker genes in **a.** scRNAseq and **b.** scATACseq datasets.

Supplementary Data 5: Global changes in chromatin accessibility between conditions at Transcription Start Site (TSS), promoters, enhancers, and DNase I hypersensitivity sites as measured by pairwise t-tests using post hoc Bonferroni correction. ns = >0.05; * $P \leq 0.05$; ** $P \leq 0.01$, *** $P \leq 0.001$; **** $P \leq 0.0001$.

	**group1**	**group2**	**n1**	**n2**	**statistic**	**Degrees of freedom**	**p**	**p.adjusted**	**p.adj.signif**
TSS_score	Control	ControlTGFB	1292	827	-22.0102	1877.351	1.06E-95	6.36E-95	****
TSS_score	Control	GSK	1292	1625	-5.45908	2745.484	5.21E-08	3.13E-07	****
TSS_score	Control	GSKTGFB	1292	4788	-3.20937	1837.72	0.001	0.006	**
TSS_score	ControlTGFB	GSK	827	1625	18.17448	1775.327	7.90E-68	4.74E-67	****
TSS_score	ControlTGFB	GSKTGFB	827	4788	24.39793	1095.379	2.38E-105	1.43E-104	****
TSS_score	GSK	GSKTGFB	1625	4788	3.80593	2522.189	0.000145	0.00087	***
TSS_score	Control	ControlTGFB	1292	827	-22.0102	1877.351	1.06E-95	6.36E-95	****
promoter_score	Control	ControlTGFB	1292	827	-22.4322	1906.185	4.28E-99	2.57E-98	****
promoter_score	Control	GSK	1292	1625	-5.09476	2744.699	3.73E-07	2.24E-06	****
promoter_score	Control	GSKTGFB	1292	4788	-3.06631	1822.224	0.002	0.012	**
promoter_score	ControlTGFB	GSK	827	1625	18.92771	1812.959	4.69E-73	2.81E-72	****
promoter_score	ControlTGFB	GSKTGFB	827	4788	25.25269	1101.523	2.23E-111	1.34E-110	****
promoter_score	GSK	GSKTGFB	1625	4788	3.490015	2498.132	0.000491	0.002946	***
enhancer_score	Control	ControlTGFB	1292	827	-2.73648	1465.278	0.006	0.036	**
enhancer_score	Control	GSK	1292	1625	9.947646	2730.284	6.31E-23	3.79E-22	****
enhancer_score	Control	GSKTGFB	1292	4788	3.332233	1890.256	0.000878	0.005268	**
enhancer_score	ControlTGFB	GSK	827	1625	10.12127	1330.572	3.01E-23	1.81E-22	****
enhancer_score	ControlTGFB	GSKTGFB	827	4788	5.328039	973.9712	1.23E-07	7.38E-07	****
enhancer_score	GSK	GSKTGFB	1625	4788	-9.64138	2631.47	1.21E-21	7.26E-21	****
DNAse_score	Control	ControlTGFB	1292	827	-24.5392	2085.948	4.88E-117	2.93E-116	****
DNAse_score	Control	GSK	1292	1625	-1.89467	2705.479	0.058	0.348	ns
DNAse_score	Control	GSKTGFB	1292	4788	-2.17128	1751.957	0.03	0.18	ns
DNAse_score	ControlTGFB	GSK	827	1625	24.66932	2096.33	3.47E-118	2.08E-117	****
DNAse_score	ControlTGFB	GSKTGFB	827	4788	30.9395	1195.165	7.20E-155	4.32E-154	****
DNAse_score	GSK	GSKTGFB	1625	4788	0.145638	2431.567	0.884	1	ns

Supplementary Data 6: Changes in chromatin accessibility between Fib 2 and MFib 1 at Transcription Start Site (TSS), promoters, enhancers, and DNase I hypersensitivity sites as measured by pairwise t-tests using post hoc Bonferroni correction. ns = >0.05; * $P \leq 0.05$; ** $P \leq 0.01$, *** $P \leq 0.001$; **** $P \leq 0.0001$.

	group1	group2	n1	n2	statistic	Degrees of freedom	p	p.adjusted	p.adj.signif
TSS_score	Fib 2_Control	MFib 1_ControlTGFB	231	214	-12.41943811	442.731142	1.36E-30	2.72E-30	****
TSS_score	MFib 1_ControlTGFB	MFib 1_GSKTGFB	214	1839	13.75785879	248.5231876	2.12E-32	4.24E-32	****
promoter_score	Fib 2_Control	MFib 1_ControlTGFB	231	214	-12.74800175	441.6966474	6.49E-32	1.30E-31	****
promoter_score	MFib 1_ControlTGFB	MFib 1_GSKTGFB	214	1839	14.35913935	249.7537118	1.70E-34	3.40E-34	****
enhancer_score	Fib 2_Control	MFib 1_ControlTGFB	231	214	3.716611109	431.169688	0.000229	0.000458	***
enhancer_score	MFib 1_ControlTGFB	MFib 1_GSKTGFB	214	1839	-2.730299054	246.8121449	0.007	0.014	**
DNAse_score	Fib 2_Control	MFib 1_ControlTGFB	231	214	-11.24945356	415.9995536	8.18E-26	1.64E-25	****
DNAse_score	MFib 1_ControlTGFB	MFib 1_GSKTGFB	214	1839	14.71322606	267.898061	2.55E-36	5.10E-36	****

a

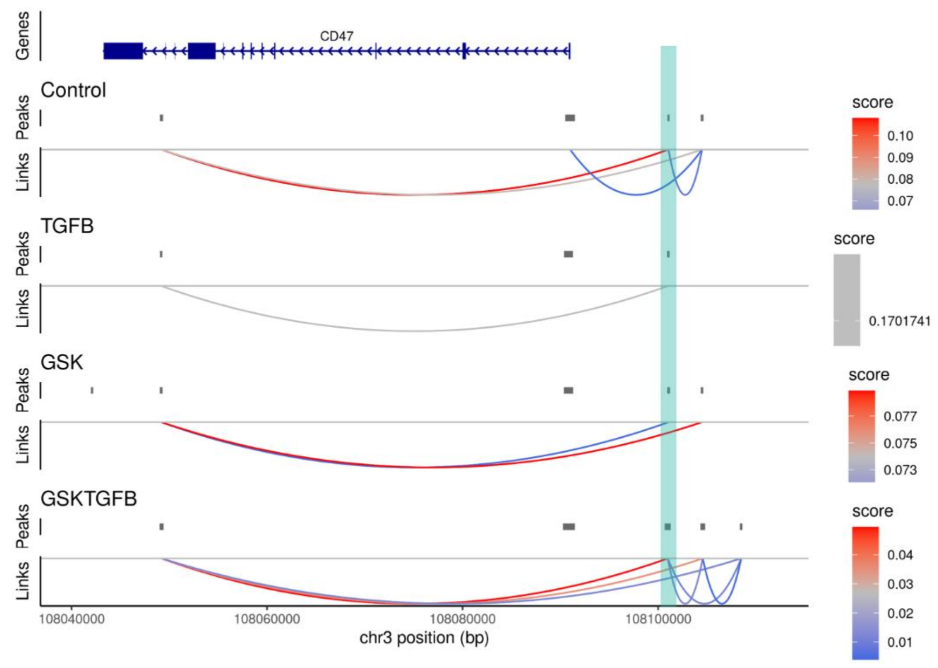
Genes
CD47
Control
Peaks
Links
TGFB
GSK
GSKTGFB
score
0.10
0.09
0.08
0.07
0.1701741
0.077
0.075
0.073
0.04
0.03
0.02
0.01
108040000
108060000
108080000
108100000
chr3 position (bp)

b

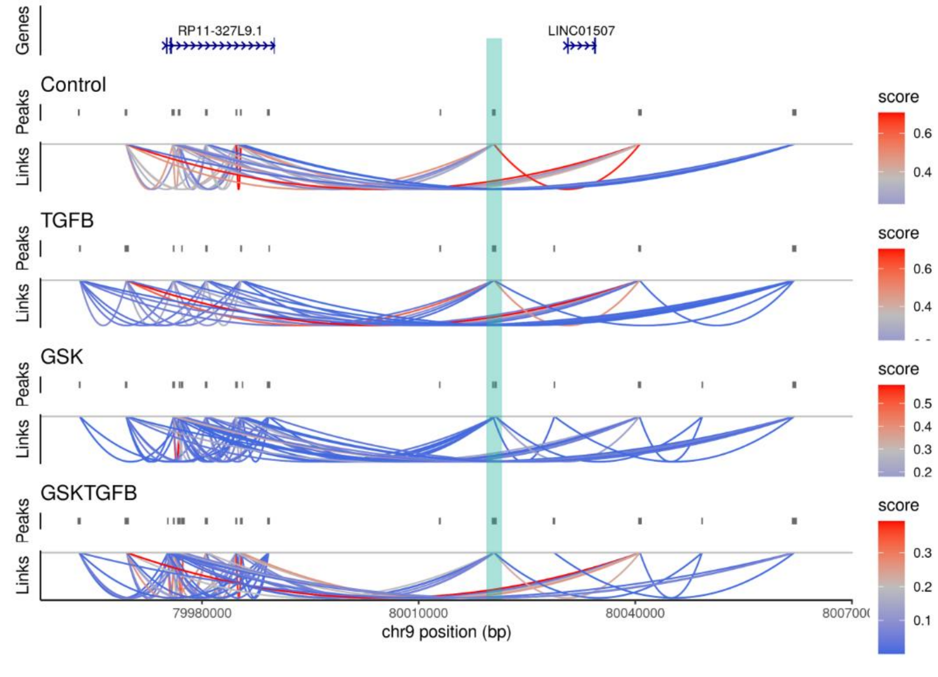
Genes
RP11-327L9.1
LINC01507
Control
Peaks
Links
TGFB
GSK
GSKTGFB
score
0.6
0.4
0.5
0.3
0.2
0.1
79980000
80010000
80040000
chr9 position (bp)

Supplementary Figure 11. Changes in cis co-accessibility observed in control, TGFβ1, and GSK343+TGFβ1 treated organoids at a putative enhancer region identified to be co-occupied by SMAD3 and EZH2 in human iPSCs and Day 7 cells.

TGFβ1 changes cis co-accessibility at a putative enhancer region in **a.** iPSCs (chr3:108090401-108091801) and **b.** Day 7 cells (chr9:80012751-80015001) compared to control or GSK343 pre-treated organoids. Cis co-accessibility links obtained from Cicero illustrate the probability of increased (red) or decreased (blue) connections between promoter and enhancer regions. Enhancer region is indicated in green.

Supplementary Data 8: Top 25 differential regulons identified in MFib 1 compared to its parent population Fib 2. Sorted on average log fold change (Avg_logFC). P_val: p-value; P_val_adj: adjusted p-value after Bonferroni correction (false discovery rate detection); Pct: percentage of cells where gene is detected in the group.

**Regulon**	**p_val**	**avg_log2FC**	**MFib 1**	**Fib 2**	**p_val_adj**
NUAK1-(+)-motif	7.82E-272	0.359355	0.996	0.93	2.07E-269
HES1-(+)-motif	1.25E-196	0.186447	0.958	0.672	3.33E-194
FOSL2-(+)-motif	4.79E-270	0.153037	1	0.989	1.27E-267
JUNB-(+)-motif	5.13E-162	0.092462	0.999	0.98	1.36E-159
BHLHE40-(+)-motif	1.41E-159	0.092391	0.888	0.479	3.73E-157
ELK3-(+)-motif	9.75E-298	0.090374	1	1	2.58E-295
GATA6-(+)-motif	5.41E-60	0.083474	0.947	0.83	1.43E-57
FOXL2-(+)-motif	6.84E-168	0.082467	0.991	0.85	1.81E-165
RARG-(+)-motif	6.55E-76	0.077709	0.773	0.435	1.74E-73
ETV3-(+)-motif	3.11E-73	0.074807	0.957	0.821	8.25E-71
CREB3L1-(+)-motif	6.58E-128	0.06672	1	1	1.74E-125
ERG-(+)-motif	4.19E-182	0.063093	1	0.996	1.11E-179
FOS-(+)-motif	1.26E-182	0.061631	1	0.934	3.33E-180
HOXA7-(+)-motif	2.97E-98	0.059534	1	1	7.88E-96
FLI1-(+)-motif	2.62E-175	0.058898	1	1	6.93E-173
KLF10-(+)-motif	8.22E-154	0.057612	1	0.989	2.18E-151
KLF2-(+)-motif	5.82E-173	0.057418	1	1	1.54E-170
XBP1-(+)-motif	2.89E-183	0.044966	1	1	7.65E-181
HOXA13-(+)-motif	7.68E-42	0.03963	0.496	0.247	2.04E-39
HIC1-(+)-motif	3.84E-49	0.037685	1	1	1.02E-46
NFIC-(+)-motif	1.39E-33	0.033138	0.702	0.514	3.67E-31
CREB3-(+)-motif	7.27E-64	0.032332	0.929	0.817	1.93E-61
JUN-(+)-motif	3.45E-200	0.031444	1	1	9.13E-198
NFIB-(+)-motif	1.64E-94	0.031232	1	1	4.36E-92
JUND-(+)-motif	4.99E-148	0.03026	1	1	1.32E-145

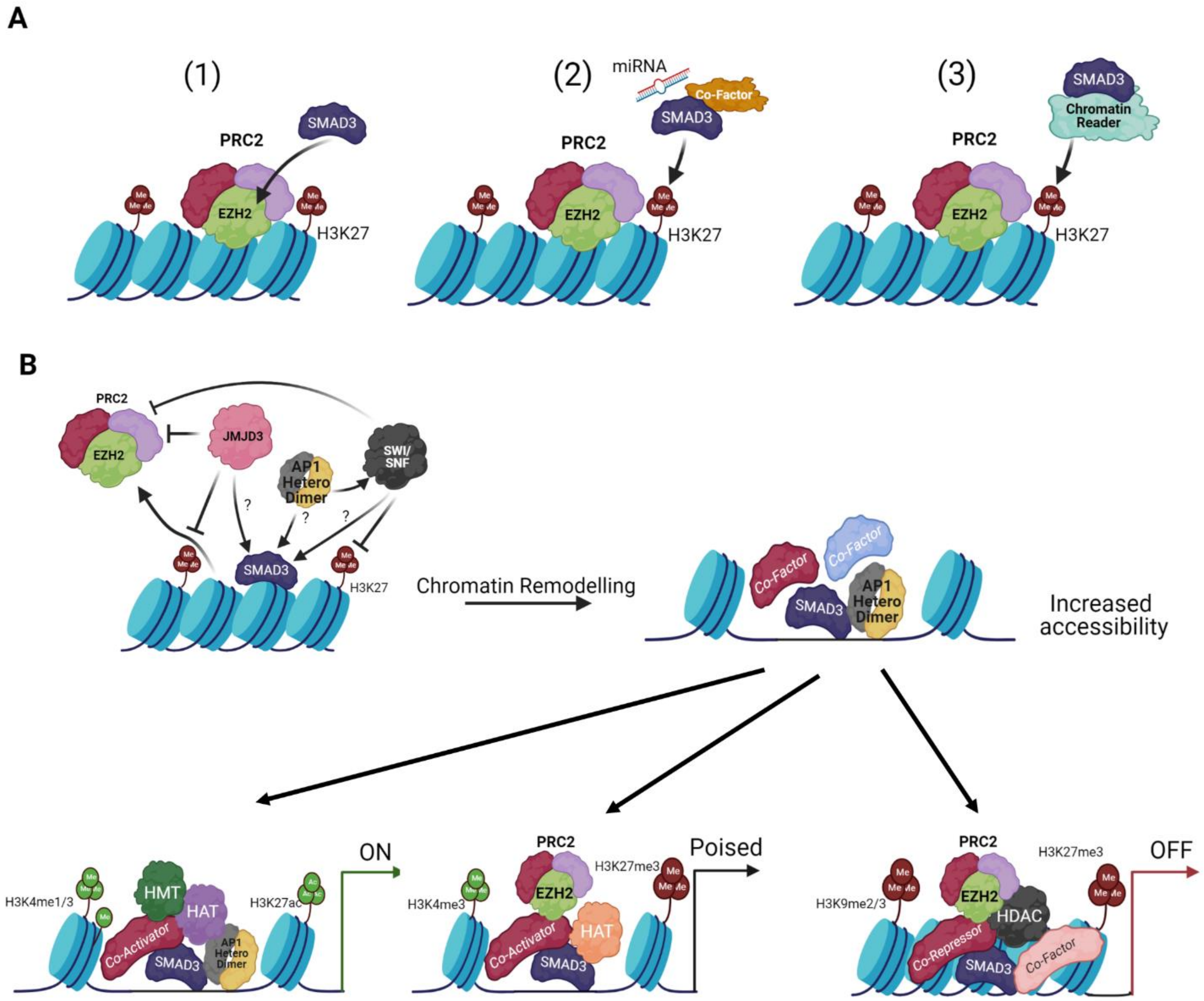

Supplementary Figure 12. SMAD3 and EZH2 cooperate to regulate chromatin accessibility

a. Schematic of TGFβ-induced increased chromatin accessibility. (1) SMAD3 directly interacts with EZH2, (2) SMAD3 indirectly interacts with EZH2, or (3) Smad3 cooperates with a chromatin reader to identify and recognise the H3K27me3 mark. **b.** Schematic showing that SMAD3 may facilitate open chromatin states by displacing the polycomb repressive complex and recruiting chromatin remodellers such as SWI/SNF and/or HDACs such as JMJD3. Interaction with AP-1 and other co-factors during chromatin remodelling may facilitate increased accessibility. To enable the activation of target gene expression, SMAD3 may recruit co-activators, histone methyltransferases, and histone acetyltransferases to catalyse the H3K4me1/3 and H3K27ac marks at enhancer regions. The recruitment of PRC2 may permit the acquisition of poised states. Finally, the recruitment of co-repressors, histone deacetylases, and the PRC2 complex may allow for the silencing of TGFβ1 target genes and the acquisition of the H3K9me2/3 and H3K27me3 histone marks. Created using Biorender.

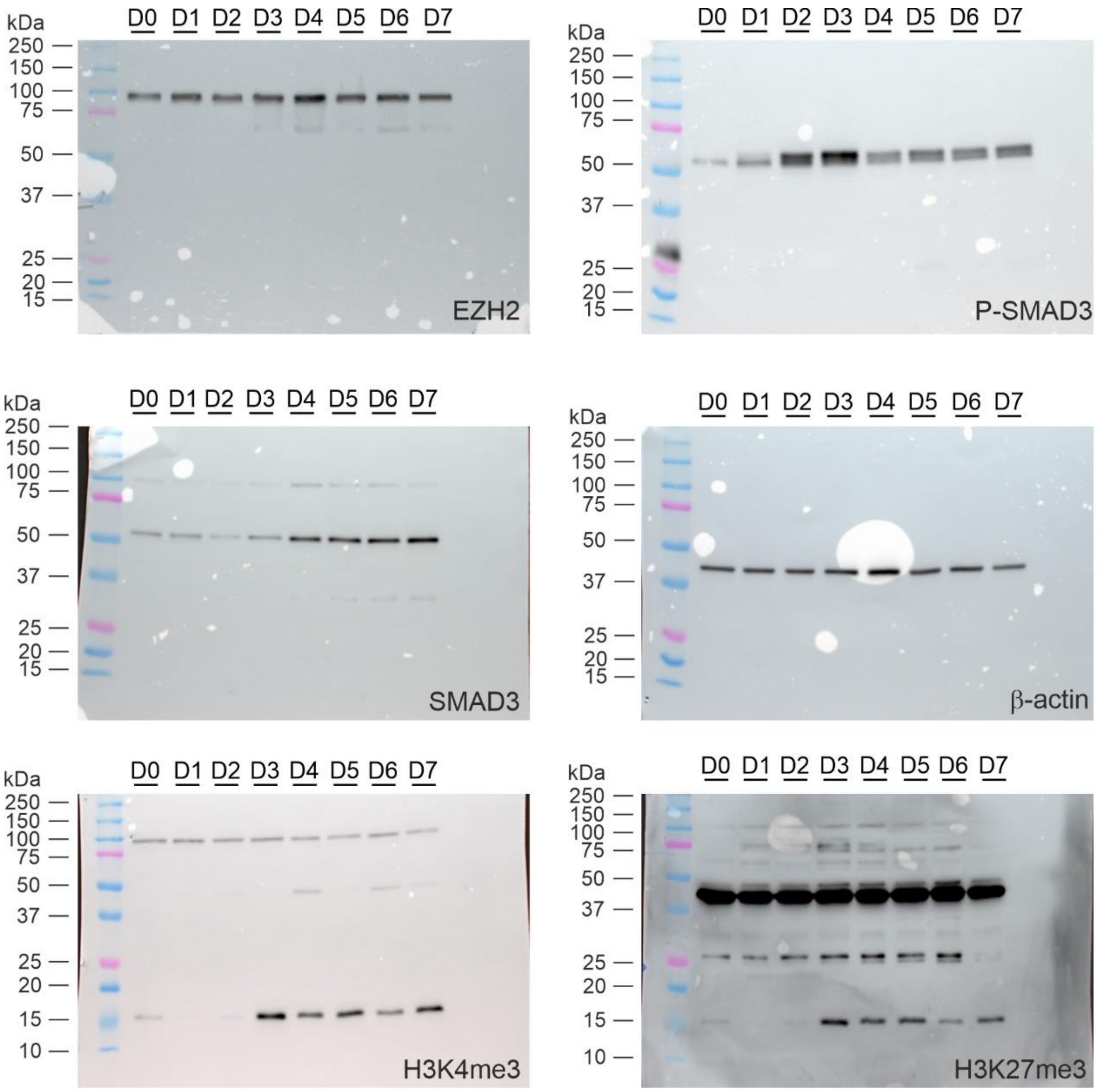

Supplementary Figure 13: Uncropped western blots used in Figure 1C.

Western blot analysis of EZH2, Phosphorylated SMAD3 (P-SMAD3), total SMAD3, β-actin, H3K4me3, and H3K27me3 expression in whole cell lysate from iPSCs over the course of nephrogenic specification.
