# Supplementary material for: Single-cell multiomics reveals the complexity of TGFβ signalling to chromatin in iPSC-derived kidney organoids
Source: Commun Biol. 2022 Nov 27;5:1301. doi: 10.1038/s42003-022-04264-1 (PMC9701233; doi:10.1038/s42003-022-04264-1)
Supplement: Supplementary file 2 — Description of Additional Supplementary Data [file 42003_2022_4264_MOESM2_ESM.pdf]

Description of Additional Supplementary Files

File name: Supplementary Data 1

Description: Marker genes used for single cell cluster annotation

File name: Supplementary Data 4

Description: Differential expression of de novo clusters Myofibroblast 1 (Cluster 2) and Stroma 1 (Cluster 3), identified in iPSC-derived kidney organoids treated with TGFβ1

File name: Supplementary Data 7

Description: Differentially accessible regions and motif enrichment in fibroblast 2 and myofibroblast 1 clusters

File name: Supplementary Data 9

Description: Source data for ChIPseq graphs (Supplementary Fig. 1A and 1B), and statistical analysis of immunofluorescence and histological images (Fig. 3e-f, 4e-f and Supplementary Fig. 8b)
