# Supplementary material for: Single-cell multiomics reveals the complexity of TGFβ signalling to chromatin in iPSC-derived kidney organoids
Source: Commun Biol. 2022 Nov 27;5:1301. doi: 10.1038/s42003-022-04264-1 (PMC9701233; doi:10.1038/s42003-022-04264-1)
Supplement: Supplementary file 7 — Reporting Summary [file 42003_2022_4264_MOESM7_ESM.pdf]

nature portfolio

Corresponding author(s): Jessica L. Davis, Ciaran Kennedy, Shane Clerkin, Niall J. Treacy, Thomas Dodd, Catherine Moss, Alison Murphy, Derek P. Brazil, Gerard Cagney Dermot F. Brougham, Rabi Murad, Darren Finlay, Kristiina Vuori, and John Crean.

Last updated by author(s): 2022/11/10

Reporting Summary

Nature Portfolio wishes to improve the reproducibility of the work that we publish. This form provides structure for consistency and transparency in reporting. For further information on Nature Portfolio policies, see our Editorial Policies and the Editorial Policy Checklist.

Statistics

For all statistical analyses, confirm that the following items are present in the figure legend, table legend, main text, or Methods section.

n/a	Confirmed	
☐	☒	The exact sample size (*n*) for each experimental group/condition, given as a discrete number and unit of measurement
☐	☒	A statement on whether measurements were taken from distinct samples or whether the same sample was measured repeatedly
☐	☒	The statistical test(s) used AND whether they are one- or two-sided<br>*Only common tests should be described solely by name; describe more complex techniques in the Methods section.*
☒	☐	A description of all covariates tested
☐	☒	A description of any assumptions or corrections, such as tests of normality and adjustment for multiple comparisons
☐	☒	A full description of the statistical parameters including central tendency (e.g. means) or other basic estimates (e.g. regression coefficient) AND variation (e.g. standard deviation) or associated estimates of uncertainty (e.g. confidence intervals)
☐	☒	For null hypothesis testing, the test statistic (e.g. *F*, *t*, *r*) with confidence intervals, effect sizes, degrees of freedom and *P* value noted<br>*Give P values as exact values whenever suitable.*
☒	☐	For Bayesian analysis, information on the choice of priors and Markov chain Monte Carlo settings
☒	☐	For hierarchical and complex designs, identification of the appropriate level for tests and full reporting of outcomes
☒	☐	Estimates of effect sizes (e.g. Cohen's *d*, Pearson's *r*), indicating how they were calculated

Our web collection on statistics for biologists contains articles on many of the points above.

Software and code

Policy information about availability of computer code

Data collection	Imaging data were collected using ZEISS ZEN 2012 (blue edition) (Zeiss Microscopy, Thornwood, NY) software installed on a ZEISS LSM800 confocal microscope or using LAS X software installed on a Leica SP8 confocal microscope. ChIP and Single cell RNA sequencing data were collected with the Illumina NextSeq control software installed on an Illumina NextSeq 500. Single cell ATAC sequencing data was collected with the Illumina NovaSeq control software installed on an Illumina NovaSeq 6000. Histological images were acquired using the EOS600D Utility software (version:2.14.20a)
Data analysis	Fiji/ImageJ (version: 2.1.0/1.53q) was used for quantification of αSMA/DAPI and POSTN/DAPI areas, and quantification of picrosirius red staining. Statistical analysis was performed in GraphPad Prism (version: 8.3.0). For scRNAseq data analysis: Cell Ranger (version: 3.1.0) was used to demultiplex, align, and generate single cell feature counts. scDblFinder was used to remove suspected doublets. Seurat (version: 3.0) was used for QC, normalisation, dimensional reduction, visualisation, clustering, and differential gene expression. Gene ontology was performed using Panther. Heat diffusion for affinity-based transition embedding (PHATE) was used to identify temporal cell trajectories and map cell fate lineages. Pseudotime trajectories were visualised using the using BBrowser software (version: 2.10). Protein-protein interaction networks were obtained using the public STRING database using Cytoscape (version 3.8.1). For scATACseq analysis: Cell Ranger ATAC (version: 1.2.0) was used to for demultiplexing and alignment. Signac(version: 1.1) was used to perform QC, generate gene activity matrix, visualization and clustering, motif analysis, differential accessibility analysis. Harmony was used for dataset integration. The Cicero package within Signac was used to construct and predict cis co-accessibility connections. Gene ontology was performed using Enrichr. SCENIC was used to determine potential regulatory transcription factors through the VSN nextflow pipeline (version: 0.26.1). For ChIPseq analysis: Illumina BaseSpace was used to align sequences to hg19 genome. Peak calling and data visualisation were performed on merged replicates using EaSeq (version: 1.111). Chromatin state segmentation was performed for iPSCs using ChromHMM from ENCODE on UCSC Genome Browser

(GRCH37/hg19). Chromatin state segmentation was performed for nephron progenitors using the 25-state chromatin model of the human foetal kidney (17 gestation weeks) epigenome (EID: E086; Donor/Sample ID: H-22676). Homer (version: 4.11) was used to perform comprehensive motif analysis. Potential enhancers were identified using human embryonic stem cell and fetal kidney data on Enhancer Atlas (version: 1.0) and GeneHancer (version 4.8). BigWig tracks for all samples were generated using usegalaxy.org . BigWig tracks were visualised using the UCSC Genome Browser.

For manuscripts utilizing custom algorithms or software that are central to the research but not yet described in published literature, software must be made available to editors and reviewers. We strongly encourage code deposition in a community repository (e.g. GitHub). See the Nature Portfolio guidelines for submitting code & software for further information.

Data

Policy information about availability of data

All manuscripts must include a data availability statement. This statement should provide the following information, where applicable:

- Accession codes, unique identifiers, or web links for publicly available datasets
- A description of any restrictions on data availability
- For clinical datasets or third party data, please ensure that the statement adheres to our policy

The datasets that support the findings of this study have been deposited at ArrayExpress with the accession codes: ChIP-seq data (Accession number: E-MTAB-10910), single-cell RNAseq data (Accession number: E-MTAB-11138) and single-cell ATAC-seq data (Accession number: E-MTAB-11139). Source data for ChIPseq graphs (Supplementary Fig. 1A and 1B), and statistical analysis of immunofluorescence and histological images (Fig. 3e, 3f, 4e, 4f and Supplementary Fig. 8b) are available in Supplementary Data 9. Source data for western blots are available in Supplementary Fig. 13. All R code used in this paper has been deposited on GitHub and is available at: https://github.com/CiaranKennedyUCD/Kidney_Organoid_Paper_scRNA_ATAC_seq (doi: 10.5281/zenodo.7311441).

Human research participants

Policy information about studies involving human research participants and Sex and Gender in Research.

Reporting on sex and gender	Not applicable
Population characteristics	Not applicable
Recruitment	Not applicable
Ethics oversight	Not applicable

Note that full information on the approval of the study protocol must also be provided in the manuscript.

Field-specific reporting

Please select the one below that is the best fit for your research. If you are not sure, read the appropriate sections before making your selection.

☒ Life sciences ☐ Behavioural & social sciences ☐ Ecological, evolutionary & environmental sciences

For a reference copy of the document with all sections, see nature.com/documents/nr-reporting-summary-flat.pdf

Life sciences study design

All studies must disclose on these points even when the disclosure is negative.

Sample size	No statistical method was used to predetermine sample size owing to the exploratory nature of the study.
Data exclusions	For single cell sequencing experiments, cells were excluded based on poor QC scores and barcode readouts that indicated empty GEMs or mulitplets.
Replication	Results are representative of the similar observations and analyses made across multiple independent experiments and technical replicates. Independent experiments were classed as monolayer differentiations or organoids derived from separate passages and/or freezebacks. Organoids used in single-cell RNA and ATAC sequencing experiments were differentiated from a single well of hiPSCs and three organoids were pooled as part of a single independent experiment and dissociated for scRNAseq and scATACseq. ChIPseq experiments included cells from two independent experiments. Organoid characterisation was performed on 3-4 organoids from a minimum of 3 independent experiments. Immunofluorescent analysis was performed on one organoid per condition, from three independent experiments (Figures 3D-F and 4D-F). Histological analysis was performed on one organoid per condition, from four independent experiments (Supplementary Figure 8).
Randomization	For all experiments, organoids of equivalent morphology were randomised and sorted into experimental groups.
Blinding	Investigators were not blinded to allocation during analyses and outcome assessment owing to the automated processes used to collect and analyse results.

Reporting for specific materials, systems and methods

We require information from authors about some types of materials, experimental systems and methods used in many studies. Here, indicate whether each material, system or method listed is relevant to your study. If you are not sure if a list item applies to your research, read the appropriate section before selecting a response.

Materials & experimental systems

n/a	Involved in the study
☐	☒ Antibodies
☐	☒ Eukaryotic cell lines
☒	☐ Palaeontology and archaeology
☒	☐ Animals and other organisms
☒	☐ Clinical data
☒	☐ Dual use research of concern

Methods

n/a	Involved in the study
☐	☒ ChIP-seq
☒	☐ Flow cytometry
☒	☐ MRI-based neuroimaging

Antibodies

Antibodies used	CDH1 (610181, mouse, monoclonal, BD Bioscience), Laminin (rabbit, polyclonal, L9393, Sigma), ZO-1 (rabbit, polyclonal, 61-7300, Invitrogen), MEIS1/2/3 (mouse, polyclonal, sc-101850, Santa Cruz), WT1 (mouse, polyclonal, sc-393498, Santa Cruz), SIX2 (11562-1-AP, Invitrogen), periostin (POSTN) (rabbit, polyclonal, PA534641, Invitrogen), PDGFRA (rabbit, polyclonal, PA516571, Invitrogen), PDGFRB (rabbit, monoclonal, 3169, Cell Signalling), and Alpha smooth muscle actin (αSMA) (A5228, Sigma)
Validation	Antibodies have been previously validated for immunofluorescence on frozen sections: CDH1 (Wang et al., 2021, PMID: 33643392), Laminin (Morizane et al., 2015, PMID: 26458176), ZO-1 (Vendor recommendation), MEIS1/2/3 (Geuens et al., 2021, PMID: 34198162), WT1 (Treacy et al., 2022 Bioactive materials, 2022 , in Press MS No. BIOMAT1160), SIX2 (Morizane et al., 2015, PMID: 26458176), periostin (Vendor recommendation), PDGFRA (Agha et al., 2020, PMID: 32613166), PDGFRB (Roswall et al., 2018, PMID: 29529015; Treacy et al., 2022 Bioactive materials, 2022 , in Press MS No. BIOMAT1160), alpha smooth muscle actin (Vendor recommendation; Kaji et al., 2020, PMID: 32501213)

Eukaryotic cell lines

Policy information about cell lines and Sex and Gender in Research

Cell line source(s)	The human iPSC line HPSI1213i-babk_2 purchased from the ECACC
Authentication	Cell line was authenticated by the ECACC (https://ebisc.org/WTSIi028-A). iPSC line was routinely checked for pluripotency and genomic integrity by immunofluorescence of pluripotency markers and genetic analysis qPCR testing.
Mycoplasma contamination	Cells were mycoplasma tested once a month using the Lonza Mycoalert detection kit and all cells tested negative.
Commonly misidentified lines (See ICLAC register)	No commonly misidentified cell lines were used.

ChIP-seq

Data deposition

☒ Confirm that both raw and final processed data have been deposited in a public database such as GEO.

☒ Confirm that you have deposited or provided access to graph files (e.g. BED files) for the called peaks.

Data access links<br>*May remain private before publication.*	https://www.ebi.ac.uk/biostudies/arrayexpress/studies/E-MTAB-10910
Files in database submission	IPSC_SMAD3_rep1, IPSC_SMAD3_rep2, IPSC_EZH2_rep1, IPSC_EZH2_rep2, IPSC_Input_1, IPSC_Input_2 ,D7_SMAD3_rep1, D7_SMAD3_rep2, D7_EZH2_rep1, D7_EZH2_rep2, D7_Input_1, D7_Input_2
Genome browser session (e.g. UCSC)	No longer applicable

Methodology

Replicates	2 biological replicates were used for each condition
Sequencing depth	All samples were sequenced to a read depth of 25 million paired end reads

Antibodies	Smad3 (ab28379, Abcam) or EZH2 (5246, Cell Signalling)
Peak calling parameters	Peak calling was performed on merged replicates using EaSeq using the adaptive local thresholding method and default settings. Peaks overlapping blacklisted features as defined by the ENCODE project were removed.
Data quality	iPSC-SMAD3 - 971 peaks<br>iPSC-EZH2 - 8528 peaks<br>D7-SMAD3 2416 peaks<br>D7-EZH2 1912 peaks
Software	Illumina BaseSpace, EaSeq, Homer, usegalaxy.org
